# ALS-linked FUS mutations dysregulate G-quadruplex-dependent liquid–liquid phase separation and liquid-to-solid transition

**DOI:** 10.1016/j.jbc.2021.101284

**Published:** 2021-10-06

**Authors:** Akira Ishiguro, Jun Lu, Daisaku Ozawa, Yoshitaka Nagai, Akira Ishihama

**Affiliations:** 1 Research Center for Micro-Nano Technology, Hosei University, Koganei, Tokyo, Japan; 2 Medical Examination Center, National Center for Global Health and Medicine, Shinjuku-ku, Tokyo, Japan; 3 Department of Neurotherapeutics, Osaka University Graduate School of Medicine, Suita, Osaka, Japan; 4 Department of Neurology, Kindai University Faculty of Medicine, Osaka-Sayama, Osaka, Japan

**Keywords:** FUS (fused in sarcoma), G-quadruplex, ALS (amyotrophic lateral sclerosis), LLPS (liquid–liquid phase separation), LST (liquid-to-solid transition), ALS, amyotrophic lateral sclerosis, βCD, β-cyclodextrin, CD, circular dichroism, FTLD, frontotemporal dementia, FUS, fused in sarcoma, LC, low-complexity, LLPS, liquid–liquid phase separation, LST, liquid-to-solid transition, NLS, nuclear localization signal, PY-CTR, C-terminal Pro/Tyr-rich region, RBP, RNA-binding protein, RNP, ribonucleoprotein, SPR, surface plasmon resonance, TEM, transmission electron microscopy

## Abstract

Amyotrophic lateral sclerosis (ALS) is a neurodegenerative disease characterized by the accumulation of protein aggregates in motor neurons. Recent discoveries of genetic mutations in ALS patients promoted research into the complex molecular mechanisms underlying ALS. FUS (fused in sarcoma) is a representative ALS-linked RNA-binding protein (RBP) that specifically recognizes G-quadruplex (G4)-DNA/RNAs. However, the effects of ALS-linked FUS mutations on the G4-RNA-binding activity and the phase behavior have never been investigated. Using the purified full-length FUS, we analyzed the molecular mechanisms of multidomain structures consisting of multiple functional modules that bind to G4. Here we succeeded to observe the liquid–liquid phase separation (LLPS) of FUS condensate formation and subsequent liquid-to-solid transition (LST) leading to the formation of FUS aggregates. This process was markedly promoted through FUS interaction with G4-RNA. To further investigate, we selected a total of eight representative ALS-linked *FUS* mutants within multidomain structures and purified these proteins. The regulation of G4-RNA-dependent LLPS and LST pathways was lost for all ALS-linked FUS mutants defective in G4-RNA recognition tested, supporting the essential role of G4-RNA in this process. Noteworthy, the P525L mutation that causes juvenile ALS exhibited the largest effect on both G4-RNA binding and FUS aggregation. The findings described herein could provide a clue to the hitherto undefined connection between protein aggregation and dysfunction of RBPs in the complex pathway of ALS pathogenesis.

Neurodegenerative disorders such as amyotrophic lateral sclerosis (ALS) and frontotemporal dementia (FTLD) are characterized by the progressive degeneration of nerve cells in the brain and spinal cord (1, 2). In these neural disorders, two RNA-binding proteins (RBPs), TDP-43 (43 kDa TAR DNA-binding protein) and FUS (fused in sarcoma), have been reported as the common causative gene products, of which modulations lead to a gain of functional toxicity or a loss of normal protein function (3, 4, 5, 6). Even though the characterization of mutants and the analysis of proteinaceous inclusions have provided important clues for elucidation how these gene products are connected with the diseases, the molecular mechanisms of ALS and FTLD remain unclear. Along this line, one important issue is that these two proteins specifically bind to mRNA containing G-quadruplex (G4) and transport to distal neurites for local translation (7, 8). Recently, we reported that ALS-linked mutations of TDP-43 are less active than wild-type in binding with G4-RNAs (9). These results suggested that the altered interaction between G4-RNAs and mutant proteins is somehow connected with the pathogenesis of ALS and FTLD.

On a noncanonical higher-order DNA/RNA structure, G4 is composed of two or more guanine tetrads, which associate through Hoogsteen hydrogen bonding leading to form a square planar structure (10, 11). G4 plays essential roles in telomere function, gene expression, and intracellular mRNA transport, and thus its dysfunction leads to cancer and neurodegenerative disorders (12). Recently, it has been anticipated that such noncanonical RNA structures are involved in the formation of RNA granules by liquid–liquid phase separation (LLPS) (13, 14). The formation of RNA granules is observed *in vitro*, but in the presence of RBPs, the functional structures are considered to be composed of ribonucleoprotein (RNP) complexes in cells (15, 16, 17). RNPs form membraneless cellular compartments and play important roles, which have been detected as germ granules and polar granules in germ cells, stress granules and P-bodies in somatic cells, and neuronal granules in neurons (13, 14, 18).

FUS is one of the most characterized RBPs known to exhibit LLPS (19, 20, 21, 22, 23, 24, 25, 26, 27). The N-terminal proximal half of FUS is composed of one QGSY-rich region and one Gly-rich domain while its C-terminal proximal half contains a single RRM (RNA recognition motif), two RGG (Arg/Gly-rich) domains interposed by a Zn finger, and a C-terminal Pro/Tyr-rich region (PY-CTR) including nuclear localization signal (NLS) (Fig. 1*A*). The N-terminal QGSY-rich region, one RGG region, and the C-terminal two RGG regions are the disordered low-complexity (LC) regions in the FUS protein sequence and are considered to mediate LLPS (25). Within the FUS protein, at least 57 residues of the N-terminal LC region between amino acids 39–95 and the multiple RNA-binding regions are both required for LLPS (24, 28). These findings altogether indicate that multiple modules of FUS influence the formation or disassembly of RNP compartments. However, the contribution of LLPS to the formation and regulation of RNP compartments carries the risk because RBPs with LC regions tend to aggregate (1, 22). Since the aberrant phase separation of G4-binding proteins such as FUS and TDP-43 is considered to link with neurodegeneration, it is critically important to understand in details how the RNA structure promotes, inhibits, and/or tunes the phase separation of associated proteins. Up to the present time, however, the molecular mechanism of the sequential phase behavior of ALS-linked FUS in LLPS and liquid-to-solid transition (LST) remains elusive.

**Figure 1:**
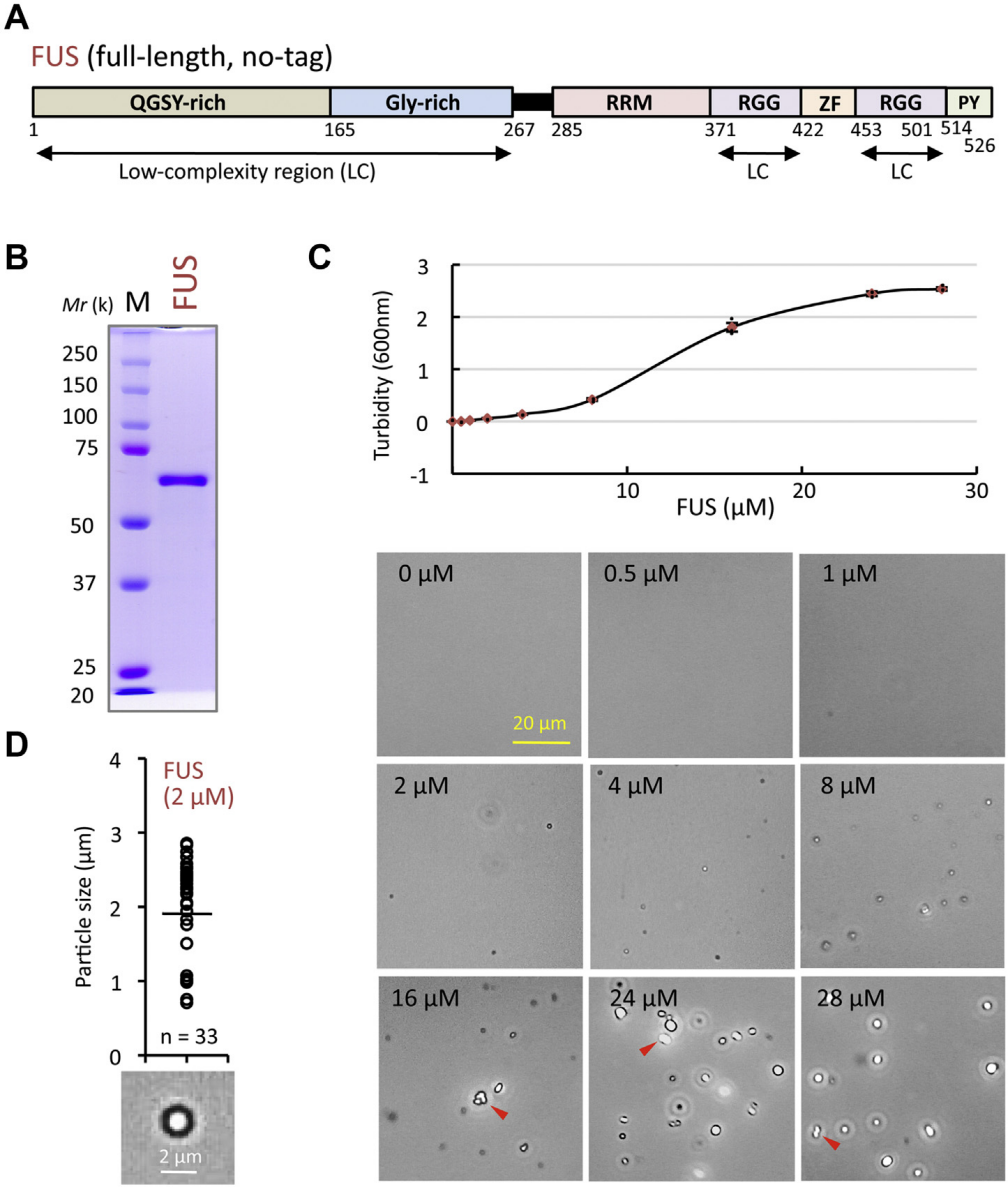
**Liquid–liquid phase separation of FUS.***A*, structural feature of FUS protein. FUS comprises of, from N- to C-terminus, one QGSY-rich region, one Gly-rich domain, one RRM, two RGG domains interspaced with a single Zn finger, and Pro-/Tyr-rich region (PY) containing NLS. Low-complexity (LC) intrinsically disordered regions are indicated below the structural map. *B*, SDS-PAGE pattern of the purified FUS protein. Two micrograms of the purified proteins was analyzed, in parallel with the marker protein mixture, by 10% SDS-PAGE, and the gel was stained with Coomassie brilliant blue. *C*, protein concentration-dependent increase of the formation of FUS condensates. The FUS condensates formed were observed by phase-contrast microscopy (scale bar, 20 μm). High-magnification images are shown in Figure S1. The *y*-axis shows the level of turbidity and the standard errors (±SEM) obtained after three independent experiments. Measurements were performed after incubating for 30 min at 25 °C. Samples of 16 μM and above were measured at 5-fold dilution. The red arrowheads indicate the condensates during fusion. *D*, average diameters of the FUS condensates formed at 2 μM FUS concentration. The size of FUS condensates was measured from the microscopic images. One typical high-magnification image is shown on the bottom.

The purpose of this study is to clarify how FUS-G4-RNA interactions contribute to LLPS and/or LST and whether ALS-linked *FUS* mutations affect their phase behavior. For this purpose, we constructed an *in vitro* system for observation of the formation and phase transition of FUS RNP condensates using the purified full-length wild-type and mutant FUS proteins and a set of FUS-binding target RNAs. Results herein described clearly indicated that FUS forms specific complexes with target RNAs in G4 structure-dependent manner and exhibits transformation through LLPS and LST pathways. In this process, multiple modules of FUS protein are involved. Noteworthy is that ALS-linked amino acid mutations gave marked alteration in the G4-dependent formation of FUS condensates and their phase separation and transition.

## Results

### Formation of the FUS condensates

In general, the phase-separating proteins with LC regions such as FUS are often aggregation-prone, and thus the purification of full-length intact proteins is difficult due to phase separation or aggregation (29). To overcome this problem, several methods have been taken by production of test proteins as fusion proteins and/or by adding macromolecular crowding agents such as dextran, poly-ethylene glycol, or Ficoll. In the case of FUS, however, the fusion proteins often exhibit a wide range of nonspecific nucleic-acid-binding ability (26, 30). In addition, the treatment of fusion proteins with protease to remove the tag protein quickly induces LLPS, and thus experiments must be conducted using mixtures of different protein states. Recently, however, we succeeded to purify the full-length tag-free human FUS in soluble form (Fig. 1*B*) (31). At the final purification step, FUS was prepared in a storage buffer containing β-cyclodextrin (βCD), a cyclic oligosaccharide, consisting of a macrocyclic ring of glucose subunits and forming hydrophobic cavities. Protein aggregation is prevented mainly through the temporary weak inclusion of the exposed aromatic residues into the hydrophobic cavity of βCD (32).

Starting from this purified FUS, we first tried to form FUS condensates lacking the surrounding membrane. The level of FUS condensate formation promoted with increase of FUS concentration (Figs. 1*C* and S1). The purified FUS at the physiological cellular concentration of 2 μM immediately formed protein condensates with an average diameter of about 2 μm, which are equal in size as intracellular condensates (Fig. 1*D*) (22, 33). Fusions of droplets were frequently observed at concentrations 16 μM and above (Figs. 1*C* and S2). Using this system of *in vitro* formation of FUS condensates, we next tried to examine possible influence of G4-RNA binding on the condensate formation.

### G4-RNA promotes the formation of FUS condensates

**Figure 2:**
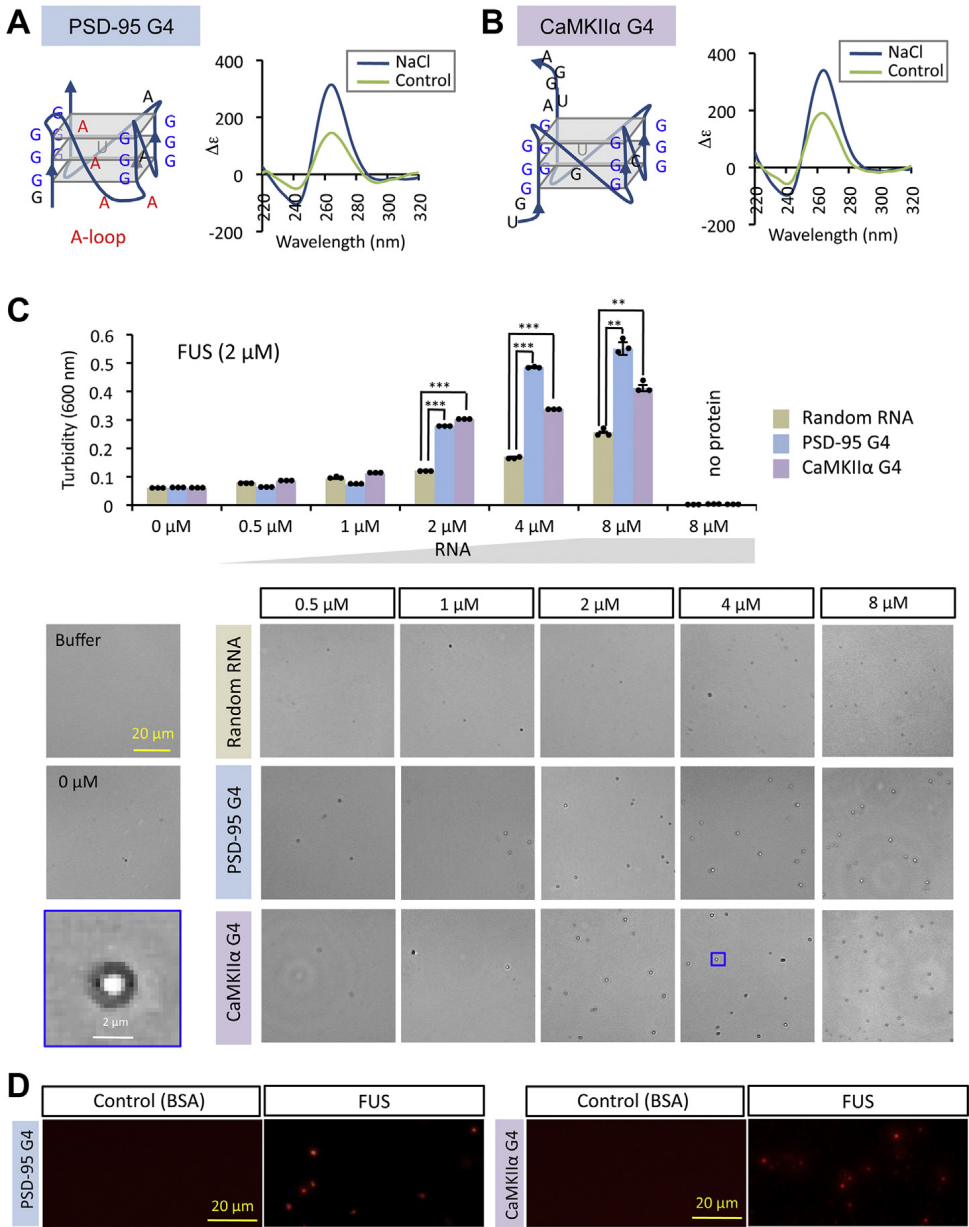
**G4-RNA promotes the formation of FUS condensates.***A* and *B*, CD spectrum of model G4-RNAs. Two species of G4-RNA (PSD-94 and CaMKIIα) were subjected to CD spectroscopic analysis in the presence (NaCl) and absence (Control) of Na^+^. The CD spectrum has a positive peak near 265 nm and a negative peak near 245 nm, both of which are characteristic of the parallel stranded G4-RNA structure. PSD-95 G4 contains four-A-loop between the first and second G triplet. *C*, influence of G4-RNAs on the formation of FUS condensates. Purified FUS protein (2 μM) was mixed with each of G4-RNAs or a reference randomized RNA (n20), and the level of FUS condensate formation was determined by measuring the turbidity. The graph shows the turbidity value of FUS solution in the presence of increasing RNA concentrations. The turbidity (y-axis) represents the average of three independent experiments together with ±SEM values. In the absence of protein, no condensate was observed. Statistical significance was determined by two-tailed Student's *t* test ∗∗*p* < 0.01, ∗∗∗*p* < 0.001. Representative images of the phase-contrast microscopy are shown for each. Scale bar represents 20 μm. *D*, FUS condensates were formed in the presence of terminal fluorescent-labeled G4 RNAs (2 μM). FUS condensates formed were observed with fluorescent microscopy. Using the same concentration of BSA (2 μM), no fluorescent signal was detected.

The purified FUS protein binds to G4-containing DNA/RNAs under structure-dependent manner and induces the deformation of G4 structure using its multiple RNA-binding modules for stable binding (31). First we confirmed the structure of G4-RNAs used for this assay using CD (circular dichroism) spectroscopy analysis. As the model G4-RNAs, we used human PSD-95 (postsynaptic density protein 95) and CaMKIIα (Ca/calmodulin-dependent protein kinase type II subunit alpha) mRNAs, which carry G4s from 3′-untranslated region of dendritic mRNAs (34). In the presence of 150 mM NaCl, both of the model G4-RNAs formed the typical parallel-stranded G4, which exhibited CD spectra with positive peak around 265 nm and negative peak around 240 nm compared in the absence of salt (Fig. 2, *A* and *B*).

We then analyzed the formation of FUS condensates in the presence of various concentrations of these model G4-RNAs. As a control, a randomized RNA (synthesized oligomer of mixed sequence composition) was used to compare and measure the formation of FUS condensates (Fig. 2*C*). Both G4 RNAs significantly promoted the formation of condensates compared with random RNA controls at concentrations 2 μM and above. No condensate formation was observed without FUS even at the highest concentrations of RNAs. Randomized RNA had a slight effect on condensate formation, supposedly due to nonspecific interactions with phosphate backbone, RNA bases of unstructured RNA, and/or G4-like sequence (35). Fluorescently labeled G4-RNAs were included in the FUS condensates, but no fluorescence signal was detected when an equal amount of BSA (bovine serum albumin) was added as a control (Fig. 2*D*). These results strongly endorse the prediction that the FUS condensates are formed through the LLPS process, and this process is promoted in the presence of G4-RNA.

**Figure 3:**
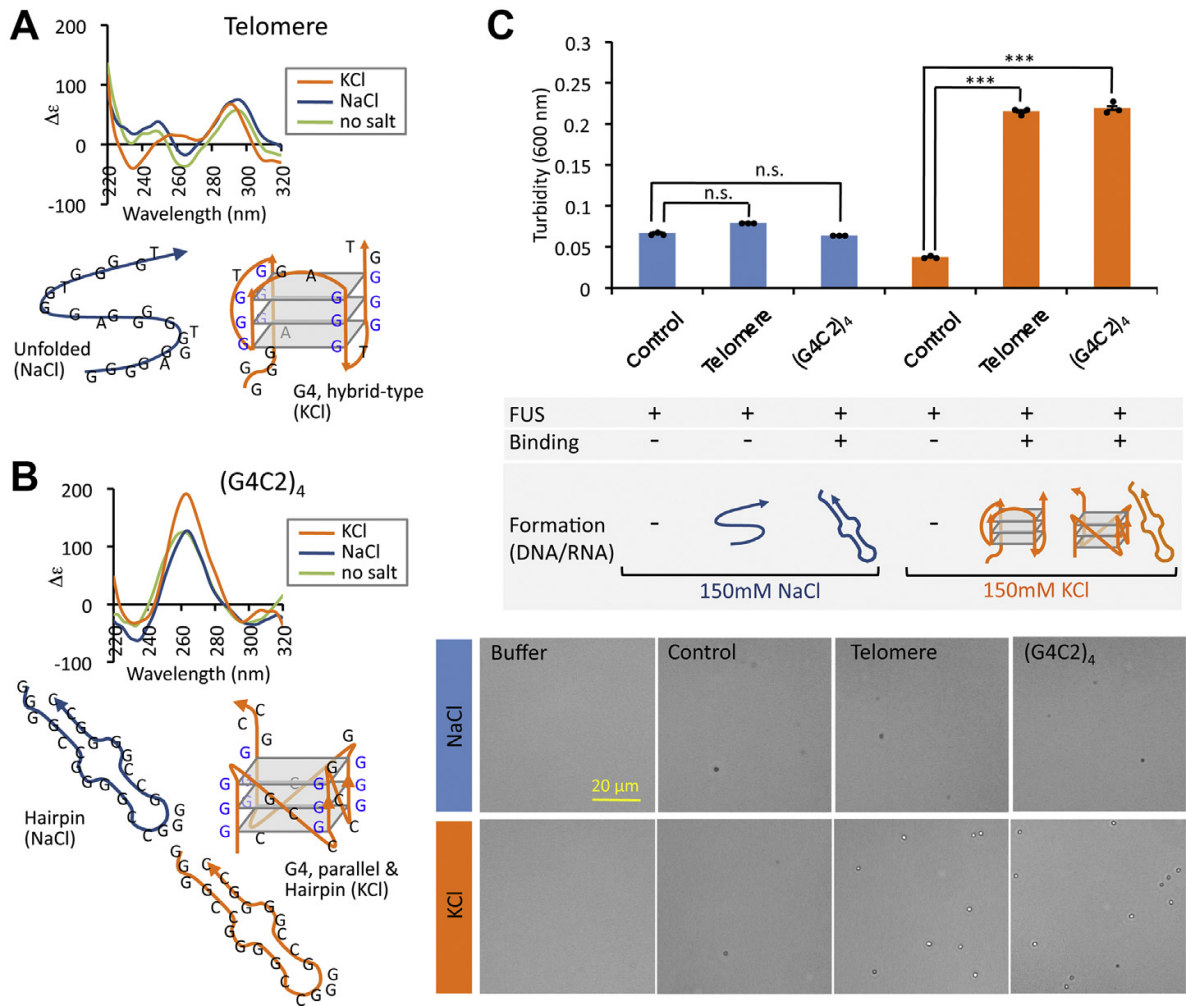
**G4 structure is required for promotion of FUS condensation.***A*, conformational alteration of telomere DNA. Telomere DNA was subjected to CD spectroscopic analysis in the presence of NaCl or KCl. Telomere DNA is known to form different conformations in the presence of KCl and NaCl (82). The CD spectrum shows a positive peak near 290 nm and a negative peak near 235 nm in the presence of 150 mM KCl, both of which are characteristic of hybrid-type G4 structure. It is, however, converted into unfolded form at 150 mM NaCl. *B*, conformational alteration of (G4C2)_4_-RNA. (G4C2)_4_-RNA exists in equilibrium between hairpin and parallel G4 conformations at 150 mM KCl, but is converted into hairpin structure at 150 mM NaCl (31, 37). *C*, G4 structure-dependent promotion of FUS condensation. The turbidity of FUS solution (2 μM) after incubation with 2 μM of telomere DNA or (G4C2)_4_. The average values of turbidity and ±SEM values were measured after three independent experiments. Statistical significance was determined by two-tailed Student's *t* test ∗∗∗*p* < 0.001. Both of telomere DNA and (G4C2)_4_ RNA gave no effect on FUS condensate formation in the buffer containing 150 mM NaCl. *Bottom panels* show representative images by phase-contrast microscopy. High-magnification images are shown in Figure S3.

To get insights into the molecular basis of LLPS promotion, we next examined whether this effect depends on either G4 structure or G-rich sequence. To discriminate these two possibilities, we performed the condensate formation assay using two well-analyzed sequences, whose structures change depending on monovalent salt species. Recently, we identified that FUS binds to human telomere DNA forming hybrid type G4 in the presence of KCl, but not to the unfolded DNA by replacing to NaCl (Fig. 3*A*; (31)). FUS also binds with both parallel-stranded G4 and G-rich hairpin structures that were formed by (G4C2)_4_, a hexanucleotide repeat expansion RNA transcribed within the ALS-related *C9orf72* gene (Fig. 3*B*; (31)). Although (G4C2)_4_ forms both structures in the presence of KCl, but only the hairpin structure by replacement of KCl to NaCl (Fig. 3*B*; (36, 37)). Neither telomere DNA nor (G4C2)_4_ affected the formation of FUS condensate in the NaCl-containing buffer (Fig. 3*C* and S3). These observations indicate that FUS binds to the hairpin structure but the FUS condensate formation is not promoted with FUS-hairpin complexes. Conversely, both telomere DNA and (G4C2)_4_ RNA significantly promoted the condensate formation of FUS in the presence of 150 mM KCl-containing buffer, in which the G4 structure is formed (Fig. 3*C*). Thus we concluded that the FUS condensation is promoted when FUS binds to G4-forming DNA/RNA.

### Influence of ALS-linked mutations on the FUS binding of G4-RNA

**Figure 4:**
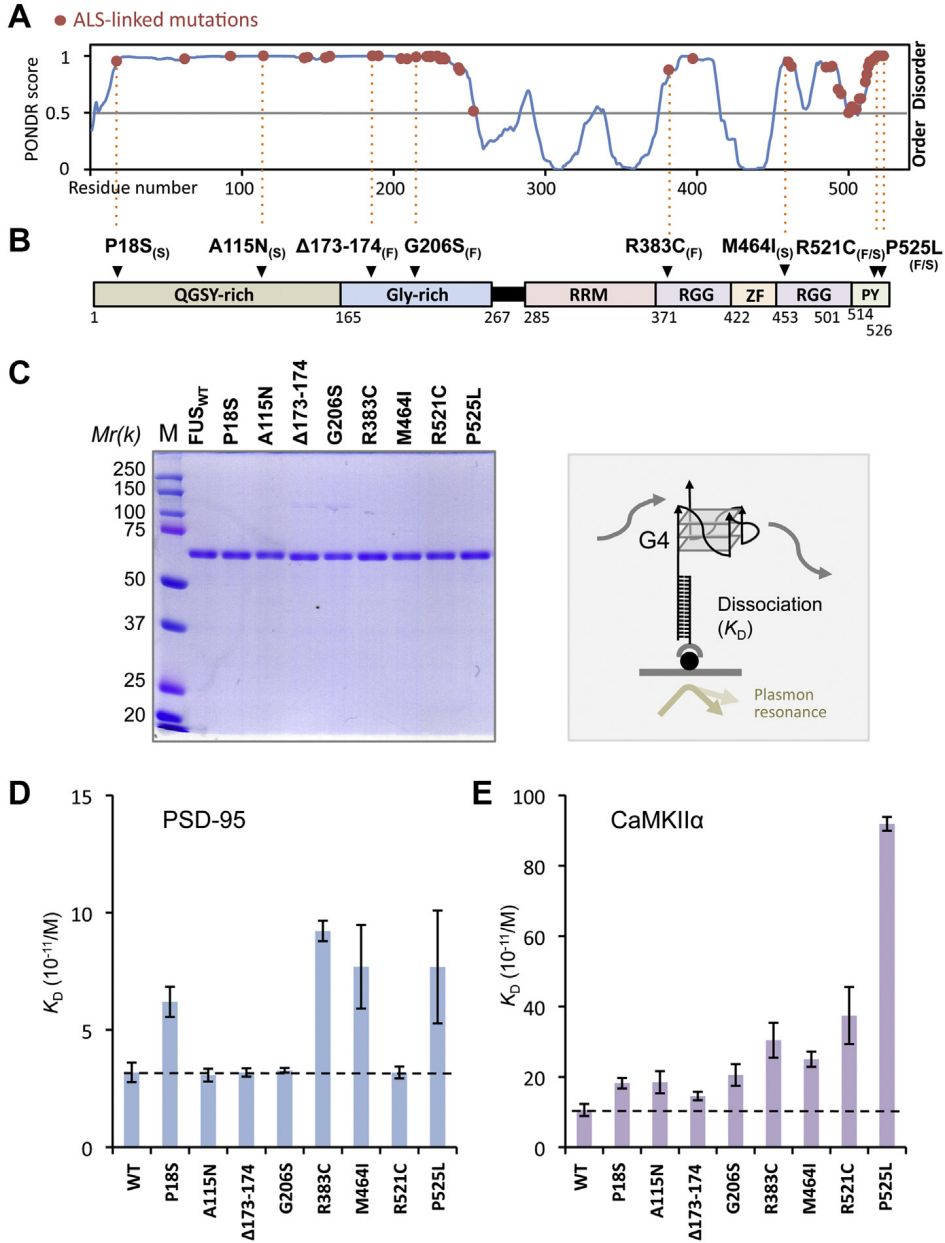
**G4-RNA-binding kinetics of eight FUS mutants.***A*, order/disorder level was calculated for FUS using PONDR algorithm (Molecular Kinetics, Washington State University). The positions of ALS-linked 56 missense and internal deletion/insertion mutations are shown along the FUS protein from N- to C-terminus (40, 41, 42). *B*, a total of eight mutants with ALS-linked familial_(F)_, sporadic_(S)_, or both_(F/S)_ at various locations on the *FUS* gene were selected and analyzed in this study. The position of each mutation is shown along the FUS map in (*A*). *C*, full-length mutant FUS proteins were purified in soluble forms according to the same procedure we developed for wild-type (31). One microgram each of the purified FUS proteins was analyzed by 10% SDS-PAGE, and the gel was stained with Coomassie brilliant blue. *Right panel* shows a schematic diagram of the SPR assay system. Terminal-biotinylated poly dT_16_ was bound to the streptavidin-coated sensor chip. Poly-dA_16_ tailed G4-RNA (20 nM) was immobilized onto the sensor chip, and various concentrations of wild-type and mutant FUS proteins were injected as the analyte. In this method, the dT_16_ oligomer was immobilized onto flow cell-2, and flow cell-1 was left blank to serve as in-line reference surface, RNA and analyte was injected to the flow cells-1 and cell-2 of the sensor chip. Plasmon resonance values (resonance unit; RU) were obtained from the flow-cell-2 data after subtracting the flow-cell-1 data. *D* and *E*, the dissociation constants (*K*_D_) of wild-type and mutant FUS proteins with PSD-95 or CaMKIIα G4-RNA. The experiments were performed three times, and the *y*-axis represents the mean ±SEM value. Kinetic data of the sensorgram are shown in Figures S4 and S5.

Up to the present time, more than 50 missense and internal deletion/insertion mutations of *FUS* have been identified in ALS patients (38, 39, 40). However, the effects of these mutations on the G4-RNA-binding activity of FUS protein and its LLPS pathway have never been investigated. These mutations are located outside the RRM and Zn finger domains (see Fig. 1*A*). With use of full-length tag-free FUS protein, we recently found the involvement of multiple structural domains of FUS other than the RRM and Zn finger domains in specific and stable interaction with highly structured DNA/RNA probes (31). Interestingly all ALS-linked missense and internal deletion/insertion mutations in *FUS* are present in the so-called disordered region (Fig. 4*A*). To get insight into possible participation of this region in the recognition and binding of G4 structure, we examined the direct binding activity of G4-RNA for the purified FUS proteins with these ALS-linked mutations. For this purpose, we selected a total of eight representative ALS-linked *FUS* mutants: two (P18S and A115N) within the N-terminal proximal QGSY-rich region; two (Δ173–174 and G206S) within the Gly-rich domain; two (R383C and M464I) within the split RGG domain; and two (R521C and P525L) within the C-terminal PY region containing nuclear localization signal (Fig. 4*B*). The full-length tag-free proteins were purified for all these eight FUS mutants as in the case of wild-type FUS protein (Fig. 4*C*).

Using the SPR (surface plasmon resonance) assay system, the kinetic parameters were measured for molecular interactions between two representative model G4-RNAs (PSD-95 and CaMKIIα) and increasing concentrations of wild-type or mutant FUS proteins. The dissociation constant (K_D_) to PSD-95 G4-RNA increased for four mutant FUS proteins, P18S, R383C, M464I, and P525L (Fig. 4*D*; for details see Fig. S4). Both R383C and M262I are located inside RGG domain, one upstream and another downstream of Zinc-finger domain, implying involvement of this RGG domain in recognition of parallel-stranded PSD-95 G4-RNA. Two mutations located near FUS terminus, P18S near N-terminal end, and P525L near C-terminal end, gave marked decrease in binding of PSD-95 G4-RNA (Fig. 4*D*; for details see Fig. S4).

To confirm this result, we also examined the binding affinity of all these eight mutant proteins to another model RNA, CaMKIIα G4-RNA. The binding affinity to CaMKIIα G4-RNA decreased for all eight FUS mutants (Fig. 4*E*; for details seeFig. S5) even though the level of reduction was variable: the reduction was the highest for P525L mutant; the reduction level was intermediate for R383C, M464I, and R521C; but only low-level reduction for other four mutants. In any case, the involvement of most FUS domains in contact with G4-RNA was suggested in agreement with the prediction that most of the FUS domains participate in binding of G4-DNA/RNA (31). The difference in binding affinity of two G4-RNAs, PSD-95 and CaMKIIα, between eight FUS mutants might be due to the difference in local structure of the G4 configuration between two G4-forming RNAs. Since PSD-95 G4-RNA contains an A-loop (see Fig. 2*A*), its G4 structure appears to be loosened or melted even at lower temperatures as detected by UV melting studies (9). In the binding of FUS to PSD-95 G4-RNA, four residues, P18, R383, M464, and P525 participate in G4 binding. In contrast, for binding to the tight G4 structure formed by G repeats in CaMKIIα G4-RNA (see Fig. 2*B*), all eight residues tested might be involved in tight G4 binding. The maximum decrease in the binding affinity to CaMKIIα G4-RNA was observed for P525L mutant at the extreme end of PY-CTR containing NLS (Fig. 4*E*; for details see Fig. S5). Thus the PY region might also participate in G4-RNA binding.

### ALS-linked FUS mutants exhibit abnormalities in the condensate formation

**Figure 5:**
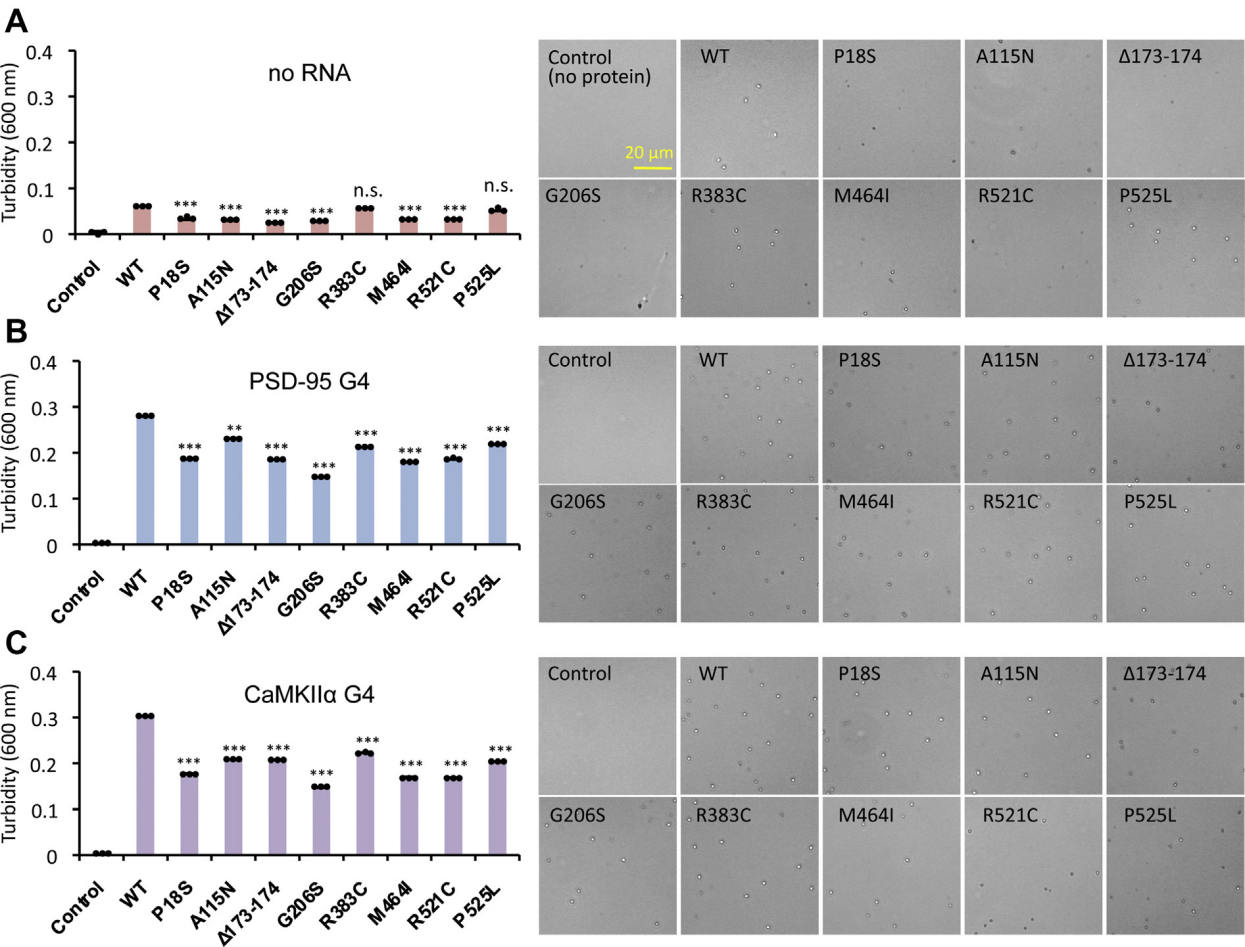
**Condensate formation of ALS-linked FUS mutants.** Purified wild-type and eight mutant FUS proteins (2 μM each) were subjected to the assay of condensate formation. *A*, turbidity level of FUS condensates in the absence of RNA. *B*, turbidity level of FUS condensates in the presence of PSD-95 G4-RNA. *C*, turbidity level of FUS condensates in the presence of CaMKIIα G4-RNA. The experiments were performed three times, and the *y*-axis represents the mean ±SEM value. Statistical significance was determined by two-tailed Student's *t* test ∗∗*p* < 0.01, ∗∗∗*p* < 0.001. *Right panels* show the representative images by phase-contrast microscopy. High-magnification images are shown in Figure S6. Particle size data of condensates are shown in Figure S7.

To get insight into the influence of ALS-linked mutations on the formation of FUS condensates through LLPS pathway, we next analyzed the effect of these mutations on the formation in the presence and absence of G4-RNAs. In the absence of RNA, wild-type FUS formed condensates as detected by the turbidity as well as the droplet level by microscopic observation (Figs. 5*A* and S6). Under the same conditions, six FUS mutants [group-1; P18S, A115N, Δ173–174, G206S, M464I, and R521C] showed decrease in condensate formation compared with wild-type FUS, but the levels of FUS condensates of two mutants [group-2; R383C and P525L] were as high as that of wild-type (Fig. 5*A*). Noteworthy is that these six positions of FUS are important for not only the condensate formation but also G4-RNA binding (see above).

In the presence of G4-RNA, however, the level of condensate formation decreased for all eight mutant proteins (Figs. 5, *B* and *C*, and S6), indicating interference of FUS condensate formation by binding with G4-RNA. The reduction of condensate formation detected by measuring the turbidity decrease is not due to reduction of the condensate size for all eight FUS mutants as detected by microscopy (Fig. S7). The reduction of condensate formation of the two group-2 mutant proteins, R383C and P525L, in the presence of G4-RNA might be due to their low binding affinity to G4-RNA (Fig. 5, *B* and *C*, and Fig. S6). In the case of four mutations [A115N, Δ173–174, G206S, and R521C], the apparent binding affinity to PSD-95 G4-RNA is the same as that of wild-type (Figs. 5*B* and S6), suggesting that the decrease in condensate formation is not simply due to defective binding to G4-RNA. Thus the ALS-linked FUS mutants all exhibited abnormalities in the condensate formation *in vitro* but its molecular basis might be due to either the alteration in binding to G4-RNA or as yet unidentified alteration in protein conformation.

### Transition of FUS condensate from liquid to solid phase

**Figure 6:**
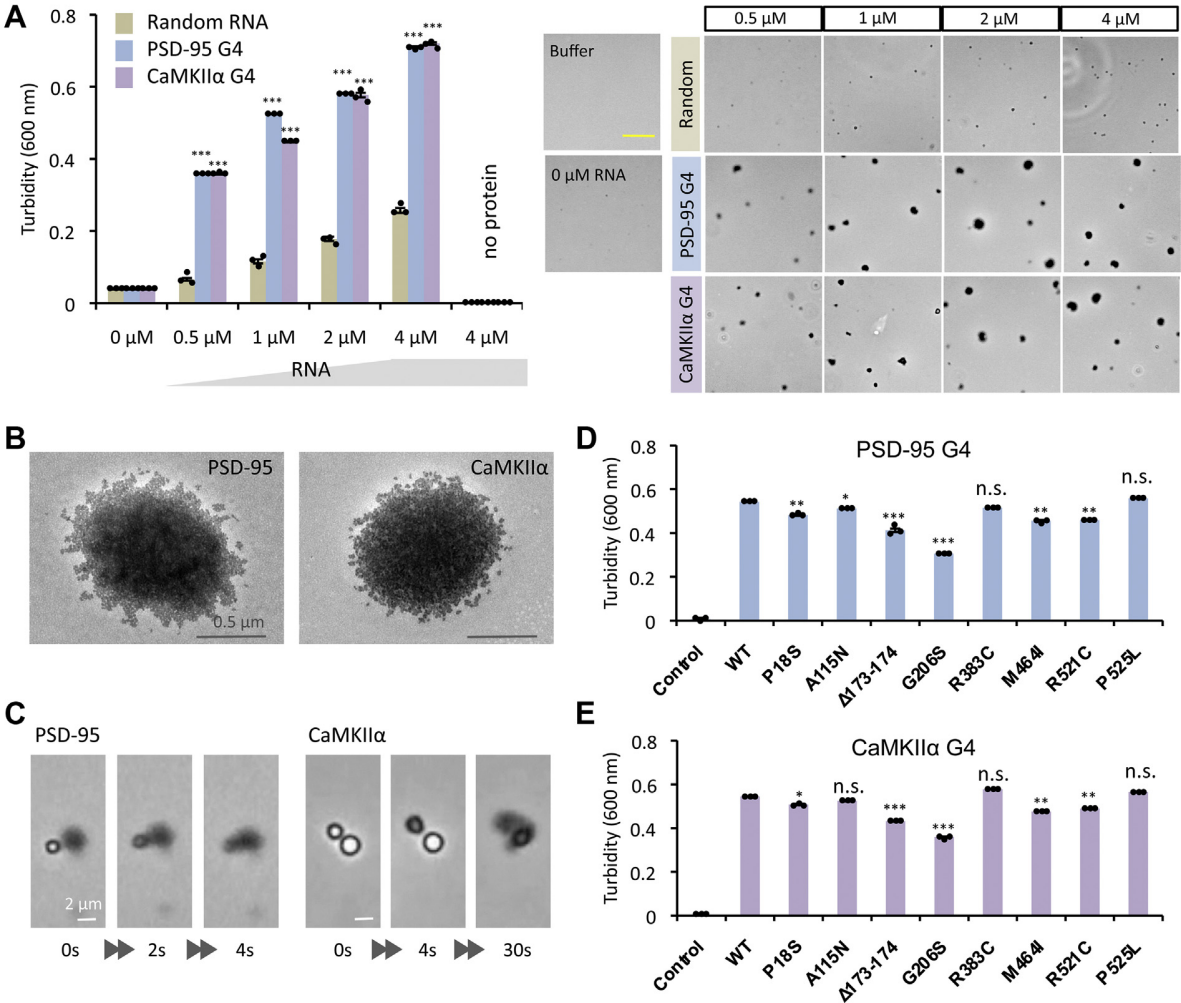
**Liquid-to-solid transition of FUS condensates.***A*, influence of G4-RNAs on the LST was measured with use of wild-type FUS protein (2 μM each) in the presence of increasing concentrations of random RNA, and PSD-95 and CaMKIIα G4-RNAs. *Right panels* show the representative images by phase-contrast microscopy. Scale bar represents 20 μm. *B*, electron micrograph of the aggregates of FUS (2 μM) formed in the presence of PSD-95 or CaMKIIα G4-RNAs (2 μM). *C*, LST of FUS condensates observed immediately after mixing in the presence of PSD-95 or CaMKIIα G4-RNAs (2 μM). Moving images are shown in Movies S2 and S3. *D*, turbidity level of FUS mutant proteins in the presence of PSD-95 G4-RNA. *E*, turbidity level of FUS mutant proteins in the presence of CaMKIIα G4-RNA. The experiments were performed in the absence of blocking DNA, and the measurements were performed after incubating for 30 min at 25 °C. The turbidity (*y*-axis) represents the average of three independent experiments together with ±SEM values. Statistical significance was determined by two-tailed Student's *t* test ∗*p* < 0.05, ∗∗*p* < 0.01, ∗∗∗*p* < 0.001. Particle size data and the representative images of mutant proteins are shown in Figure S10.

We then followed the fate of FUS condensates in the presence of RNAs. The FUS condensates remained stable for a while after addition of G4-RNA, but the condensates were gradually broken and instead protein aggregates appeared after 4 h (Fig. S8). Since these solid-like aggregates did not arise directly in the solution of purified FUS protein alone, it might arise *via* liquid condensates (or condensate droplets). To block nonspecific protein interactions, plasmid DNA was added into the assay of condensate formation at relevant concentrations (for details, see Experimental procedures). Even if the DNA is removed from the reaction mixture, condensate droplets are formed through LLPS pathway. In the absence of the DNA, the condensates were unstable, and the transition from the condensate droplets to the solid aggregates took place rapidly. The conversion always took place *via* the condensate droplet, and thus G4-RNAs promoted this process of FUS aggregation through LLPS pathway (Fig. 6*A*). This LST process is very rapid as observed by moving image (Movie S1), finally yielding mossy aggregates (Fig. 6, *A* and *B*) similar to the previous observations *in vitro* and *in vivo* (41, 42, 43). In the middle of this G4-RNA-dependent reaction, the condensate droplet collided with the FUS aggregates, leading to yield larger aggregates (Fig. 6, *A* and *C*, and Movie S2 and S3). The aggregate was further confirmed to be in a solid-phase state because it was resistant to 1,6-hexanediol, an agent known to disrupt liquid-phase condensates (Fig. S9) (44, 45). This transition was not observed in the absence of RNA or with the addition of randomized RNA (Figs. 6*A*, and S10*A*).

To confirm the causal relationship between the phase transition of FUS condensates and the ALS-linked mutations, we next analyzed the LST of FUS condensates for all eight mutants in the presence of G4-RNAs (Fig. 6, *D* and *E*, and Fig. S10, *B* and *C*). The six group-1 mutants [P18S, A115N, Δ173–174, G206, M464I, and R521C] showed a decrease in the transition level compared with wild-type FUS, while the level of two group-2 mutations [R383C and P525L] was as high as that of wild-type FUS. This finding was unexpected because these two mutations reduced the formation of condensate droplets in the presence of G4-RNAs (see Fig. 5, *B* and *D*). The group-2 P525L mutation affects the structure of PY-NLS (46), thereby leading to alter the rate of condensate formation. Likewise, the group-2 R383C mutation within the RGG region may also play an important role in FUS structure needed for protein–protein interactions in the solid phase. In contrast, group-1 mutations do not appear to exacerbate aggregation due to the reducing LLPS pathway.

### Structure–function relationship of FUS mutants

**Figure 7:**
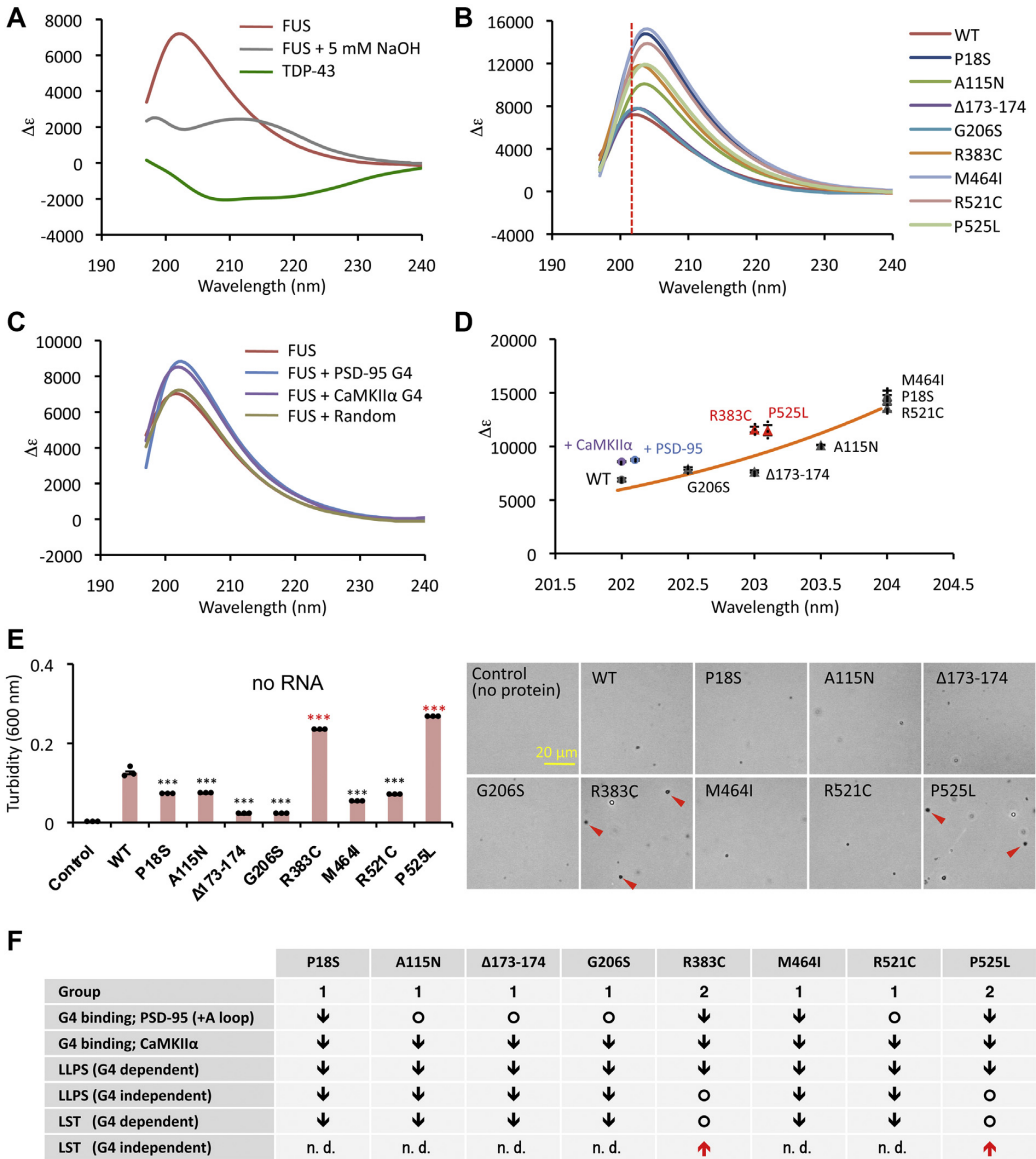
**Classification of ALS-linked mutations.***A*, far-UV CD spectrum of 0.2 μM FUS in the absence and presence of NaOH. Spectrum of full-length tag-free TDP-43 under the same assay conditions is indicated as a control. *B*, CD spectrum of ALS-linked eight mutant FUS proteins. *C*, detection of conformational alteration of 0.2 μM FUS in the presence of 0.2 μM G4 RNAs, after incubating for 30 min at 25 °C. CD spectra values were subtracted by each RNA spectra (For RNA spectra see Fig. S11*A*). *D*, scatter plot of each peak of FUS and mutant proteins from (*B*). *Circle* indicates peak value of FUS wild-type, and *triangles* indicate peak values of eight mutant proteins. Peak values of after mixing of FUS wild-type and G4-RNAs are also shown (+PSD-95 and + CaMKIIα). The *orange line* represents the exponential approximation curve of FUS wild-type and mutant proteins. The averages and ±SEM for three independent experiments are indicated. *E*, turbidity level of FUS condensates formed in the absence of RNA. The experiments and data analysis were performed as described in Figure 6. *Right panels* show the representative images by phase-contrast microscopy. *Red arrowheads* indicate samples that showed aggregate formation. Particle size data are shown in Figure S11*B*. *F*, classification of ALS-linked mutations in G4 binding. Characteristic variations are compared with the wild-type: *Down arrows* indicate decrease; *upward arrows* indicate increase; *circles*, no change. n.d., not detected.

FUS has been thought to form parallel cross-beta structures (19, 24, 47). In order to understand its conformational alteration generated by ALS-linked FUS mutations, we performed secondary structure estimation using CD spectroscopy. The CD spectrum of wild-type FUS exhibited strong positive peak at nearly 202 nm, which implied the enrichment of parallel β-sheet confirmation (Fig. 7*A*) (48). In the presence of NaOH, the peak decreased due to structural destruction (Fig. 7*A*). In comparison with FUS, human full-length TDP-43 possessed a typical α-helix confirmation with a negative peak at 206 nm under the same buffer condition as previously identified (49). The positive peaks of all mutant proteins shifted to the longer wavelength side compared with the wild-type, which is generally a characteristic of shifting to β-turns, and the peak intensity also increased (Fig. 7*B*). These mutations might cause changes in β-sheet and β-turn occupancy (50, 51).

The two group-2 mutants, R383C and P525L, that did not affect the LST in the presence of G4-RNA (see Fig. 6), showed essentially the same spectra (Fig. 7*B*). These two mutant proteins [R383C and P525L] appear to have anomalous structures and different properties from group-1 mutations. Besides, we confirmed conformational alteration of FUS by G4-RNA binding. FUS-G4-RNA complexes exhibited more emphasized positive peak around at same wavelength (Figs. 7*C* and S11*A*), suggesting that the binding enhanced the stability of the β-sheet state involved in the FUS-FUS association essential for LLPS. In contrast, randomized RNA had no apparent effect on the FUS conformation. The scatter plot graph indicates the peak values of each CD spectrum of FUS mutants and their exponential approximation curve (see Fig. 6*D*). Group-2 peaks are significantly separated from the fitting curve, and structural changes closer to the enhanced β-sheet state may facilitate the FUS-FUS association. This hypothesis is supported by the results that group-2 mutant proteins avoided reduction at LLPS levels (see Fig. 5*A*) and LST levels (see Fig. 6, *D* and *E*). However, in the absence of G4-RNA, the mimetic structure state is expected to be less stable, due to the lack of enhancing effect by G4.

For instance, the P525L mutant protein easily aggregated under the physiological conditions (43, 52, 53). The formation of mutant FUS condensates was then examined in the absence of blocking DNA. Although, the turbidity of the group-1 was significantly suppressed, group-2 proteins were more turbid than the wild-type, forming some aggregated masses in the absence of G4-RNA, which were not observed for the wild-type FUS (Fig. 7, *E* and *F*). These anomalous molecular characteristics of group-2 might be related to the functional toxicity of these mutants. Moreover, the P525L mutation is also associated with altered nuclear localization, which may have a serious impact on the pathogenesis of ALS. In fact, this P525L mutation is known to be associated with severe clinical course in juvenile ALS patients (54, 55). Along this line, it could be worthwhile to note the synergistic effect of inhibition of Karyopherin β2 (46) and G4-dependent phase behavior in disease development. Although R383C and P525L mutations tended to promote the conversion of FUS condensate droplet to solid phase, this pathway was not identified in group-1 mutations (Fig. 7*E*). Similarly, *in vivo* analysis of humanized mutant FUS mice with the group-1 mutation (R521C) revealed a decrease in local protein synthesis without cytoplasmic aggregation (56). In conclusion, we predict that the six group-1 mutations bring on the loss of normal function of FUS. On the other hand, the group-2 R383C and P525L mutations also impose a possible gain of toxic function in the pathogenic mechanism *via* accelerating the formation of inclusion bodies.

## Discussion

### Role of G4-RNA in LLPS and LST of FUS protein

Previously, we demonstrated that FUS-RNA binding is extremely dependent on the RNA conformation (31). Some of the mRNAs that are transported to the distal areas for local translation have more specific functions than general localization signals (57). In this study, we employed to a simple *in vitro* model of FUS condensate that allows us to analyze the underlying molecular interactions between G4 and FUS that promote specific condensation. Thereby we observed the effect of G4-RNA association on the formation of FUS condensates and found, for the first time, the acceleration of FUS condensate formation by G4-RNA. Our observations revealed that FUS forms specific complexes with target RNAs and exhibits transformation through LLPS and LST in G4 structure-dependent manner. We also examined the direct binding activity of G4-RNA for a total of eight FUS proteins with ALS-linked mutations and found that the binding affinity was reduced for all of them. Its binding properties are important factors to consider when studying the FUS function and complex molecular mechanism underlying ALS. In particular, ALS-associated RBPs, FUS, TDP-43, hnRNPA1 (heterogeneous nuclear ribonucleoprotein A1), hnRNP A2/B1, hnRNPA3, EWSR1 (Ewing's sarcoma RNA binding protein 1), and TIA1 (T cell-restricted intracellular antigen-1) have been suggested to exhibit RNP granules mediated by LLPS (28, 33, 47, 58, 59, 60, 61, 62). Surprisingly, all these proteins recognize and bind to the G4-RNA (8, 63, 64, 65, 66, 67), but the participation of G4 structure in the LLPS pathway remains unidentified. All of these proteins are known as aggregation-prone with LC regions, but we have succeeded in purifying TDP-43 and FUS untagged full-length proteins in soluble forms (8, 31).

RNP granule-like assemblies precipitated from the brain tissue and cultured cell extracts by biotinylated isoxazole (b-isox) are rich in LC sequences (19). The specific RNAs could be required to form the granule-like assembly in the cellular stress response (68, 69). The distribution of mRNA molecules in RNP granules is calculated to be about 10% of the intracellular mRNA molecules (70). Dendritic mRNAs that are believed to enter neuronal RNP granules for dendritic translocation, encoding PSD-95, CaMKIIα, MAP2 (microtubule-associated protein 2), Arc (activity-regulated cytoskeleton-associated protein), Shank1 (SH3 and multiple ankyrin repeat domains 1), Shank3, GluR1 (glutamate receptor 1), FMR1 (fragile X mental retardation 1), and Dendrin, were observed to be enriched by b-isox precipitation (20). Interestingly, all these mRNAs have one or more G4-forming sequences in the untranslated region (34, 71).

As noted in this report, mutant studies indicated the participation of disordered LC regions of FUS in both the formation of FUS condensates through LLPS pathway and the following LST from the condensate droplet to the solid aggregate state. Here the LLPS and LST pathways could be directly observed by phase-contrast microscopy and moving images. The enhancement of these processes by G4-RNA might be attributable to the conversion of disordered state of LC to a stable state driving these transitions. The disordered LC regions might undergo conformational changes and become ordered state upon binding with its partner RNA (72). FUS is known to undergo reentrant LLPS with promiscuous RNAs by charge reversal, and the properties of condensates are dependent on the stoichiometry (33, 73). However, we have found no evidence of the reentrant LLPS by progressively higher G4 RNA ratio (see Fig. 2*C*). Our results appear to be consistent with recent report that RNAs of 30 nucleotides or less do not bind to multiple FUS proteins (27). This particular length appears to be required, which is reasonable occupancy as FUS contains multiple units of RNA-binding modules (31, 74). In fact, the dissolution of FUS condensate, which was not seen with the short randomized RNA (20 nt) (see Fig. 2*C*), was observed with the long randomized RNA (68 nt) (Fig. S12). Therefore, our results do not show dynamic formation and dissolution of FUS droplets (75), which could be due to G4 promote LLPS with properties different from complex coacervation. Previous report has shown that GFP-fused FUS protein promotes droplet formation by TERRA (telomere repeat containing RNA) G4-RNA (33). The dissolution of the droplets may be due to an interaction different from what we have observed, or the GFP tag may be affecting it. The binding of G4-RNA on to the LC regions induces the cation–π interactions between the cationic and aromatic residues of FUS, leading to drive FUS-mediated LLPS pathway (76, 77, 78). G4 is composed of guanine tetrads and stabilized by π-π stacking (79). We have recently found that FUS binds to G4 and alters its conformation (31). It is plausible that the π-π stacked guanine tetrads are destructed and provide more π interactions during condensate formation, resulting in additional protein–RNA or RNA-RNA interfaces. The recognition of G4 structure enhances the formation of FUS condensate, but simple G-rich sequence does not induce this LLPS process. Therefore, FUS is able to bind to G-rich RNA hairpin structure (31), (74) but this binding completely loses the ability to enhance the condensate formation (see Fig. 3). Many RBPs bind to unstructured RNAs, but some require higher-order RNA structures for molecular organization by which particular functions in mRNA destiny. The mechanism herein proposed may also operate for other ALS responsible G4-binding proteins. The finding of G4-RNA-depending formation of FUS aggregates through the LLPS and LST pathways could provide new strategies to conquer ALS and drug development.

## Experimental procedures

### Plasmids

*E. coli* expression plasmid pET-FUS was described previously (31). *E. coli* expression plasmids for mutant FUS proteins (pET-FUS_P18S_, pET-FUS_A115N_, pET-FUS_Δ173-174_, pET-FUS_G206S_, pET-FUS_R383C_, pET-FUS_M464I_, pET-FUS_R521C_, and pET-FUS_P525L_) were all constructed in this study by PCR-based site-directed mutagenesis method using a set of primer pairs (Table S1).

### Proteins

Full-length tag-free human wild-type FUS protein and its mutant proteins were overproduced in *E. coli* and purified as described previously (31). The purified FUS proteins (0.5, 1, or 7 mg/ml) in the storage buffer (10% glycerol, 20 mM HEPES [4-(2-hydroxyethyl)-1-piperazineethanesulfonic acid]-NaOH, pH 6.8, 300 mM NaCl, 0.1 mM EDTA [ethylenediaminetetraacetic acid], and 10 mM β-cyclodextrin) were stored frozen at –80 °C. Full-length tag-free human TDP-43 protein was overproduced in *E. coli* and purified as described previously (8).

### *In vitro* liquid–liquid phase separation and liquid-to-solid transition assays

For liquid-like condensate formation, stock FUS proteins were diluted in the reaction buffer (20 mM PIPES [1,4-piperazinediethanesulfonic acid sesquisodium salt]-NaOH, pH 6.8, 0.8 mM MgCl_2_, 1.8 mM CaCl_2_, and 150 mM NaCl or KCl, at final concentrations), to which 0.1 mg/ml plasmid DNA (pCMVtag2A; Agilent Technologies) or various concentrations of RNAs were added to make in a final volume of 80 μl (Reactions containing plasmid DNA: Figs. 1, *C* and *D*, 2, *C* and *D*, 3*C*, 5, S2, S3, S6–S8, and S12). By diluting the storage buffer, the purified FUS immediately forms a protein condensate at the physiological cellular concentration of 2 μM. To observe the LST, plasmid DNA was removed from the reaction solution (Reactions without plasmid DNA: Figs. 6, 7*E*, S9, S10, and S11*D*). Turbidity measurements and photographs were taken after incubating for 30 min at 25 °C using a spectrophotometer (Eppendorf BioPhotomelor pus), a phase-contrast microscopy (Olympus CX42), and a fluorescent microscopy (Olympus BX53). Particle size was counted using ImageJ software (80). 1,6-Hexanediol sensitivity analysis was performed using wild-type FUS (2 μM) and two indicated G4-RNAs (2 μM) (Fig. S9). The reactions were incubated in the presence or absence of blocking DNA for 30 min at 25 °C as in standard method, then 1,6-hexanediol was added and observed 15 min later as described previously (45). Phase separation assay with FUS and the long promiscuous RNA was performed using wild-type FUS (2 μM) and various concentrations of long randomized RNA (68 nt) (8), and turbidity was measured after incubating for 30 min at 25 °C.

### Circular dichroism (CD) spectra analysis

The structure of DNA/RNA was confirmed by CD spectrum analysis using 1 μM RNA in the buffer used for the phase separation assay with or without monovalent salts at 25 °C using a CD spectrometer (Jasco J-820) as described previously (8). For conformational analysis of wild-type and mutant FUS proteins (0.5 mg/ml), the purified protein samples were diluted in PBS (phosphate-buffered saline) to make 0.2 μM solution and then subjected to CD analysis in the presence or absence of 0.2 μM RNAs (Table S1). Under the adjusted same conditions, TDP-43 (0.2 μM) was analyzed as a control.

### Surface plasmon resonance (SPR) analysis

SPR analysis was performed as described previously (8) in the SPR buffers (20 mM PIPES-NaOH, pH 6.8 at 25 °C, 0.8 mM MgCl_2_, 0.05% Tween-20, and 150 mM NaCl) using a BIAcoreJ instrument (Sytiva) at 25 °C. Kinetic constants of RNA–protein interaction were calculated using BIAEVALUATION software version 3.0 (Sytiva) according to the manufacturer's instructions. The poly-dA_16_ tailed oligonucleotides used in this study were obtained from GeneDesign, Inc. (RNAs) and Fasmac Co., Ltd (Random RNA and DNAs).

### Transmission electron microscopy (TEM)

TEM analysis was performed as described previously (81). Ten microliters of reaction sample was adsorbed onto carbon film-coated 400-mesh copper grids (Nisshin EM). Fixing the grid by inverse forceps, 1% (w/v) phosphotungstic acid (pH 7.0) was added to the grid for 60 s and the grid was dried. Samples were then imaged using a Hitachi H-7650 TEM at an acceleration voltage of 80 kV.

## Data availability

All data are in the manuscript and supporting information or are available upon request from the authors: Akira Ishiguro (akira.ishiguro.iu@hosei.ac.jp) at Hosei University.

## Supporting information

This article contains supporting information.

## Conflict of interest

The authors declare that they have no conflict of interests with the content of this article.

## Supporting information

Movie S3

Legend of Movie S1–S3
