# Supplementary material for: ALS-linked FUS mutations dysregulate G-quadruplex-dependent liquid–liquid phase separation and liquid-to-solid transition
Source: J Biol Chem. 2021 Oct 6;297(5):101284. doi: 10.1016/j.jbc.2021.101284 (PMC8567205; doi:10.1016/j.jbc.2021.101284)
Supplement: Figures S1–S12 and Table S1 [file mmc1.pdf]

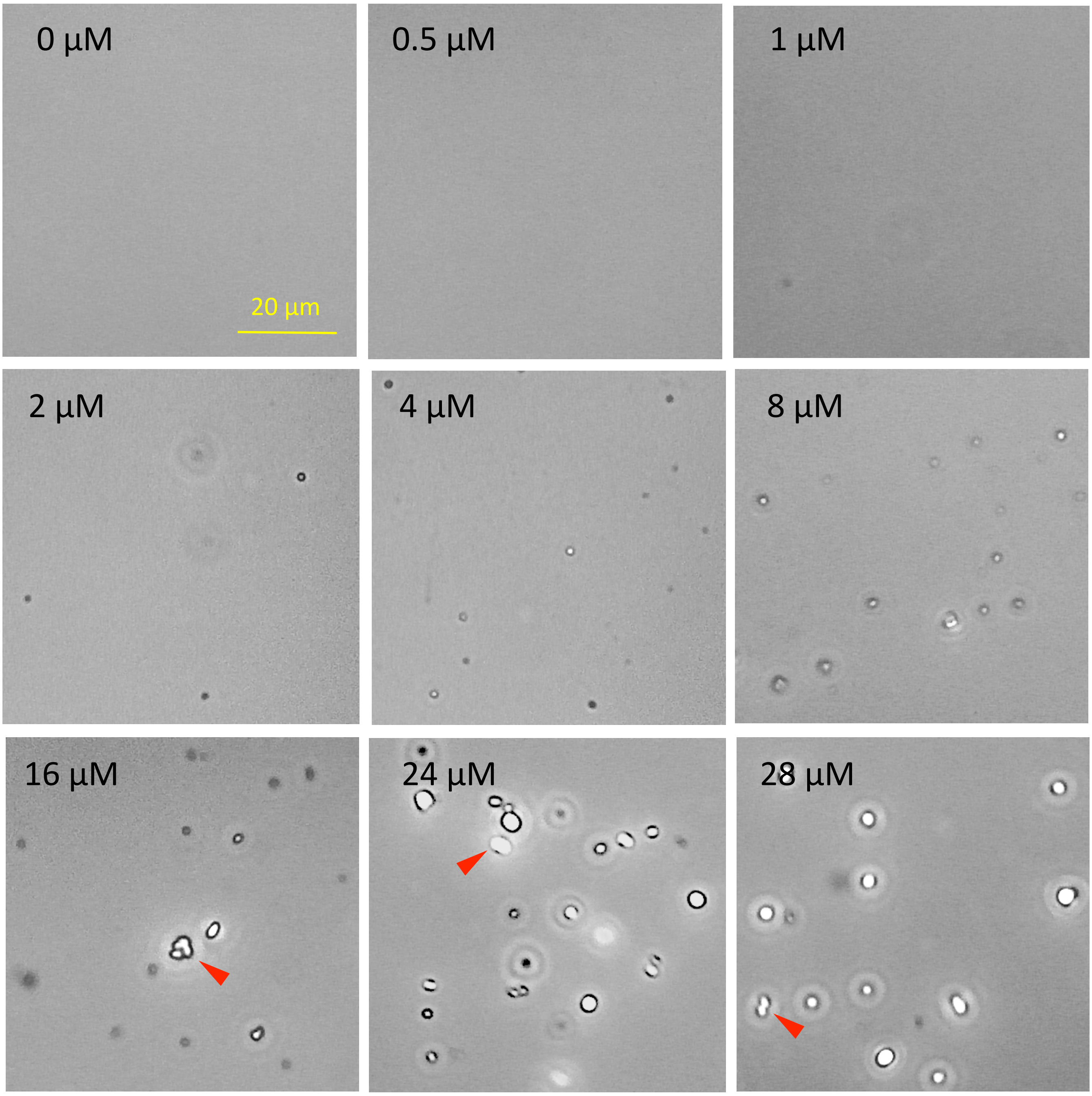

Figure S1. Concentration-dependent LLPS. [Related to Figure 1C]
Protein concentration-dependent increase of the formation of FUS condensates.

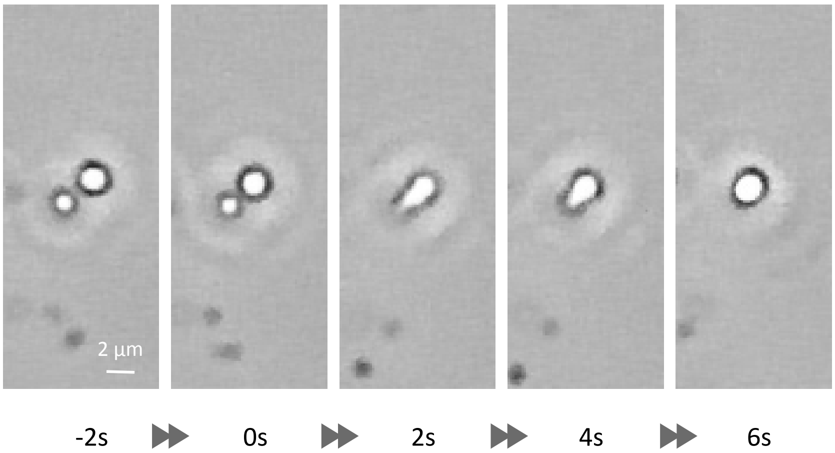

Figure S2. Liquidity of FUS condensates. [Related to Figure 1C]
The liquidity of FUS condensate was confirmed by fusion experiments at a protein concentration of 24 µM.

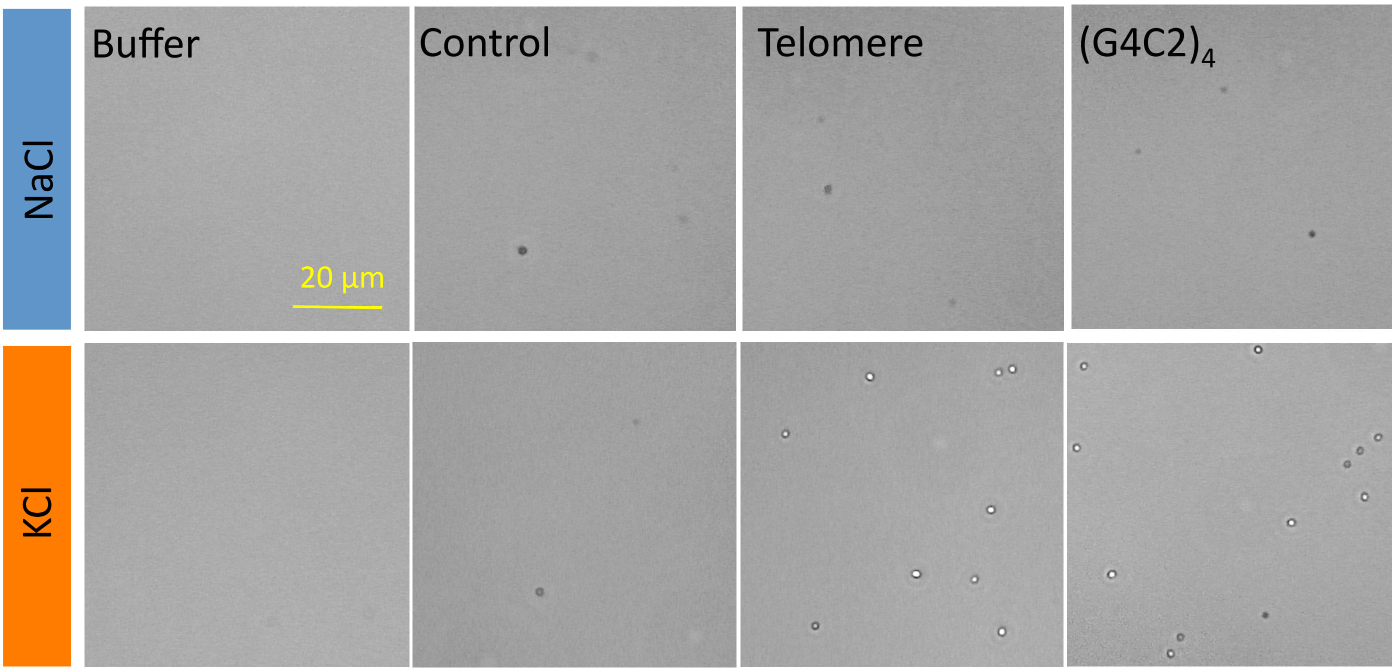

Figure S3. G4 structure is required for promotion of condensation. [Related to Figure 3C]
G4 structure dependent promotion of FUS condensation.

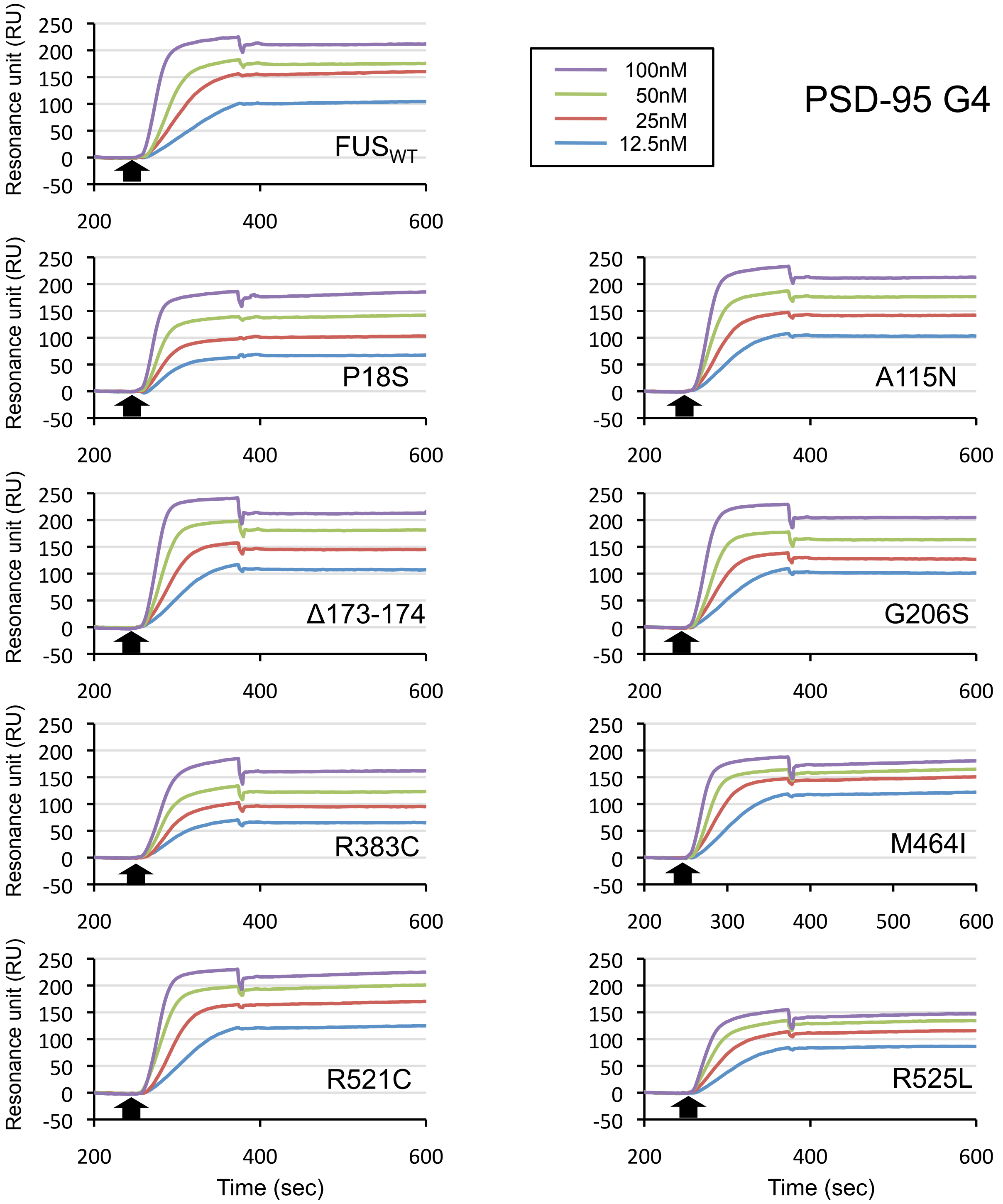

	WT	P18S	A115N	Δ173-174	G206S	R383C	M464I	R521C	R525L
K_A (10^9/M)	31.7±4.4	16.2±1.7	32.7±3.0	31.5±1.8	30.5±0.9	10.9±0.5	13.4±2.7	31.5±2.6	11.1±8.3
K_D (10^{-11}/M)	3.2±0.4	6.2±0.6	3.1±0.3	3.2±0.2	3.3±0.1	9.2±0.4	7.7±1.8	3.2±0.3	7.7±2.4

Figure S4. SPR sensorgram of the interaction between FUS mutant proteins and PSD-95 G4-RNA [Related to Figure 4]. A total of 8 mutant FUS proteins were subjected to the SPR assay for detection of interaction with PSD-95 G4-RNA (20 nM). Various concentrations of wild-type or each of the mutant FUS proteins were injected into G4-RNA immobilized flow-cell with dissociation times of 250 s (indicated by arrow). Each representative sensorgram shows the association phase and the dissociation phase in a magnified. Kinetic constants were calculated from the SPR sensorgrams, and are shown at the bottom. The calculation was performed with a 1:1 binding model. Values represent the mean ± SEM of three independent experiments.

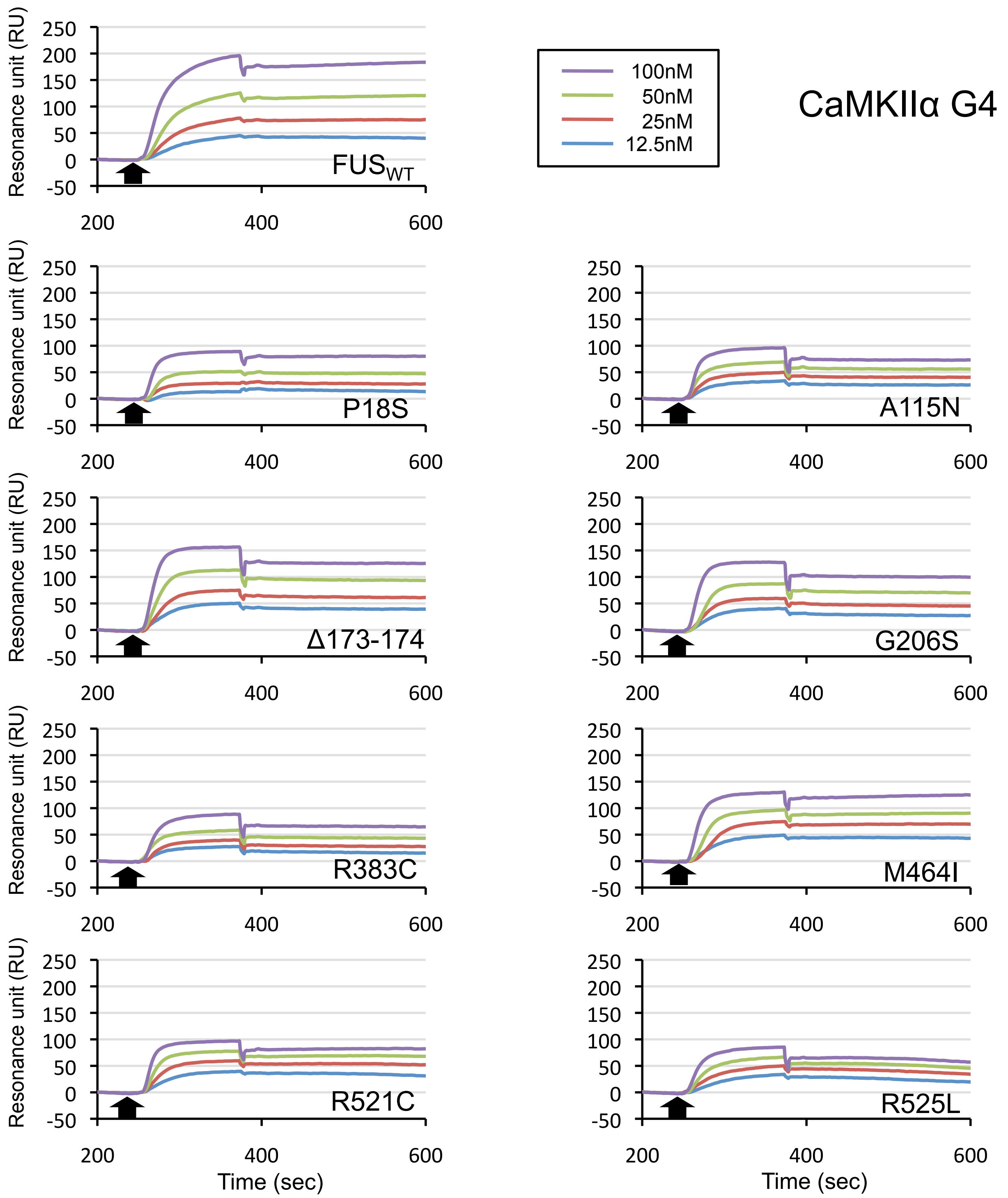

	WT	P18S	A115N	Δ173-174	G206S	R383C	M464I	R521C	R525L
K_A (10^9/M)	9.6±1.7	5.5±0.5	5.5±0.9	6.9±0.5	5.0±0.8	3.3±0.5	4.0±0.3	2.7±0.5	1.1±0.0
K_D (10^{-11}/M)	10.6±1.7	18.2±1.5	18.5±3.2	14.5±1.2	20.5±3.1	30.4±5.0	25.0±2.2	37.4±8.1	91.9±2.0

Figure S5. SPR sensorgram of the interaction between FUS mutant proteins and CaMKIIα G4-RNA [Related to Figure 4]. The SPR assay was performed essentially the same experimental protocol as in Figure S1 except that CaMKIIα G4-RNA was used in place of PSD-95 G4-RNA.

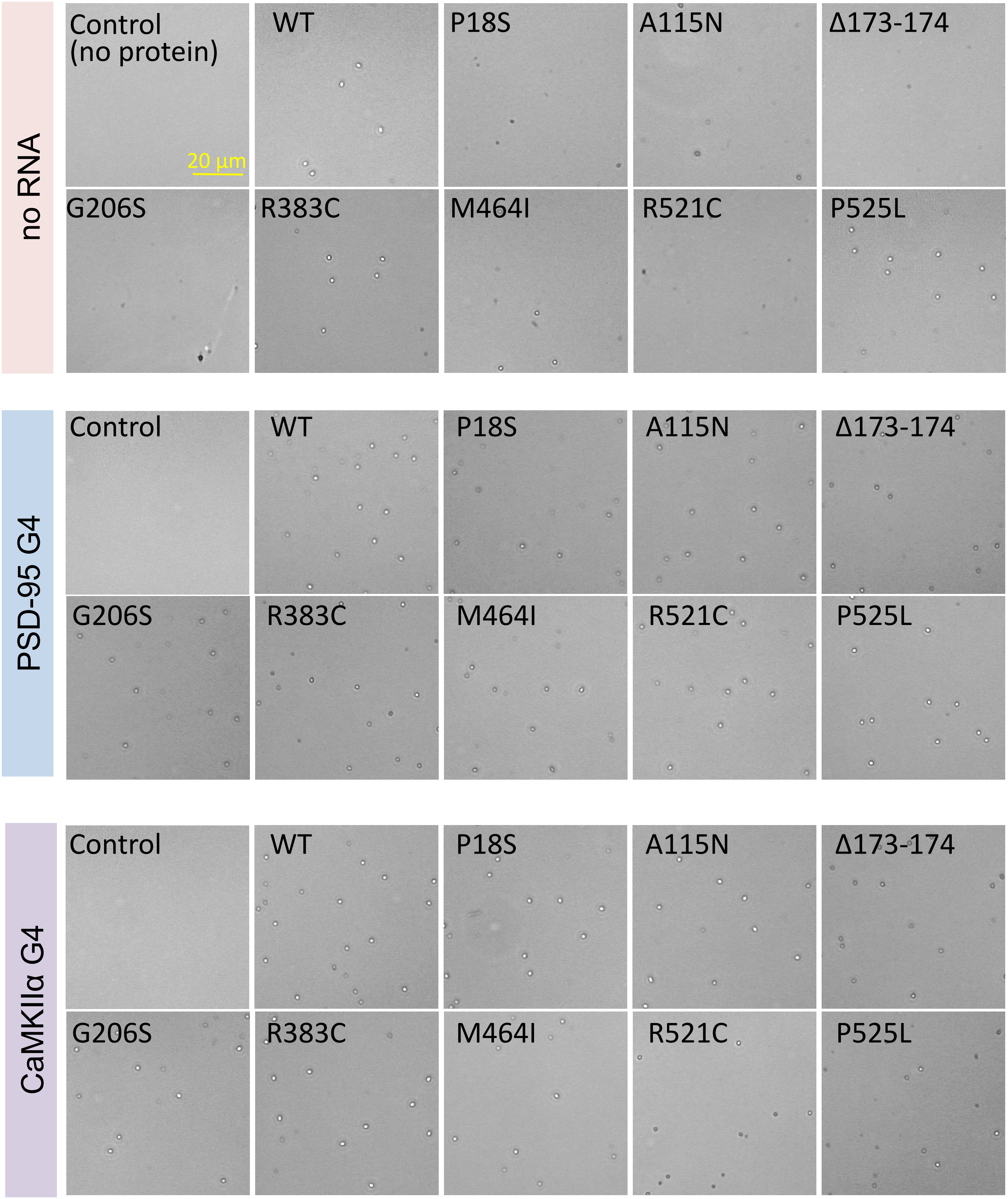

Figure S6. Condensate formation of ALS-linked FUS mutants. [Related to Figure 5]
A, Turbidity level of FUS condensates in the absence of RNA. *B,* Turbidity level of FUS condensates in the presence of PSD-95 G4-RNA. *C,* Turbidity level of FUS condensates in the presence of CaMKIIα G4-RNA.

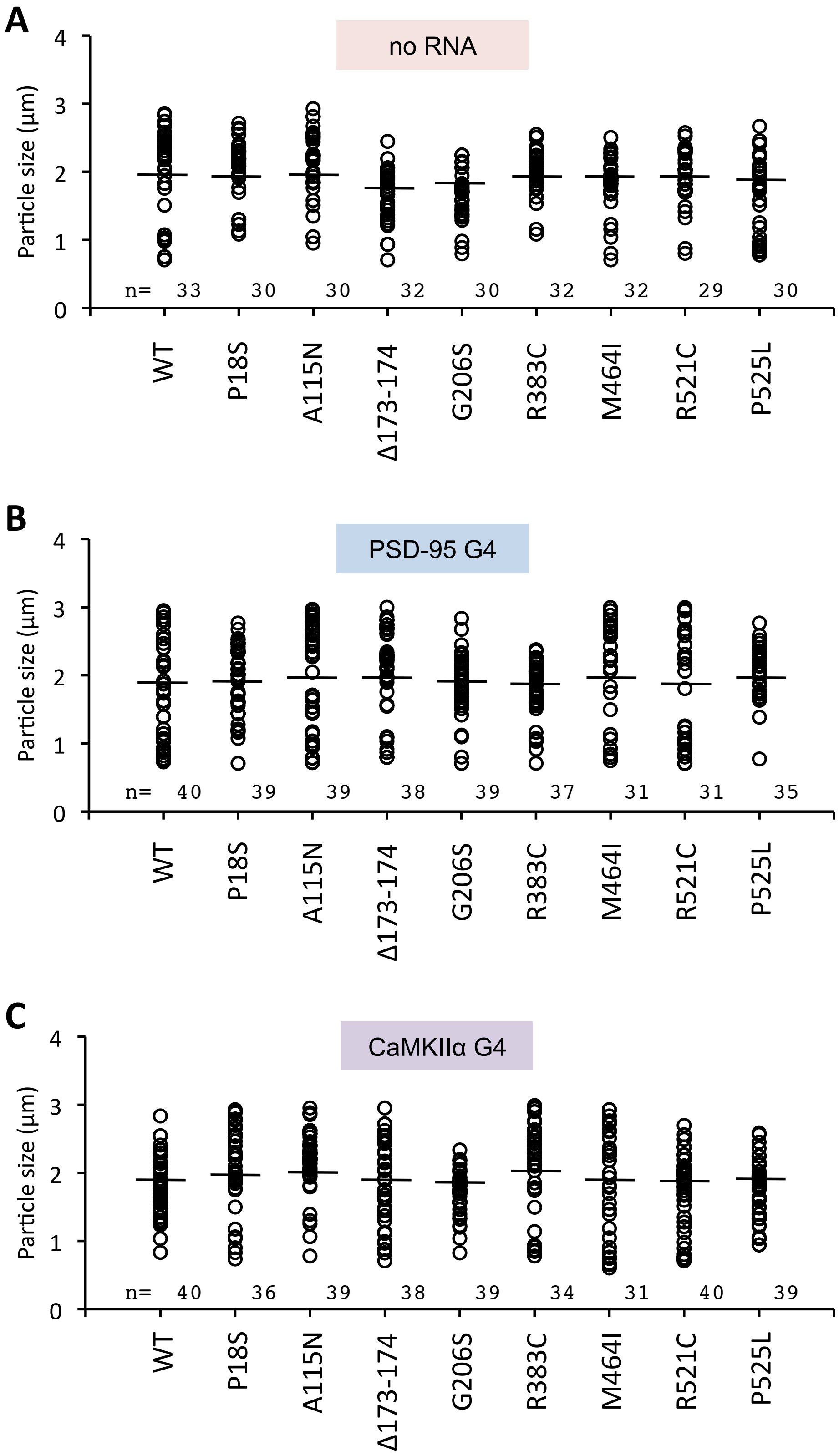

Figure S7. Particle size of FUS condensates [Related to Figure 5].
A, Particle size of wild-type and mutant FUS condensates (2 µM) formed in the absence of RNA. *B*, Particle size of wild-type and mutant FUS condensates formed in the presence of 2 µM PSD-95 G4-RNA. *C*, Particle size of wild-type and mutant FUS condensates formed in the presence of 2 µM CaMKIIα G4-RNA. The horizontal line shows the average value.

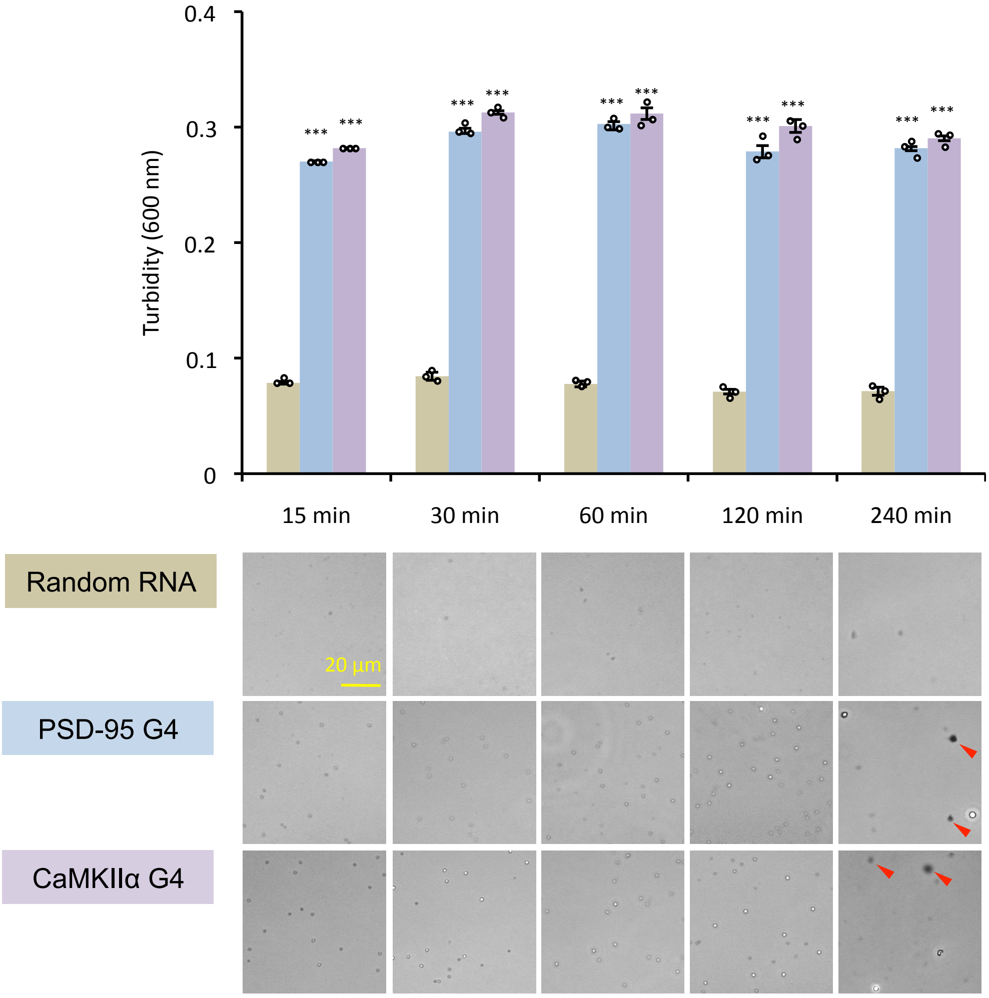

Figure. S8 Time course of the formation of FUS condensates.
Wild-type FUS protein (2 μM) was mixed with random RNA, PSD-95 G4-RNA or CaMKIIα G4-RNAs. After incubation for 15, 30, 60, 120 and 240 min, the turbidity was measured to determine the level of FUS condensate formation. The experiments were performed three times, and the *y*-axis represents the mean ±SEM value. Statistical significance was determined by two-tailed Student's t-test ***P < 0.001. Bottom panels show representative images by phase contrast microscopy. Red arrowheads are indicting aggregate formations.

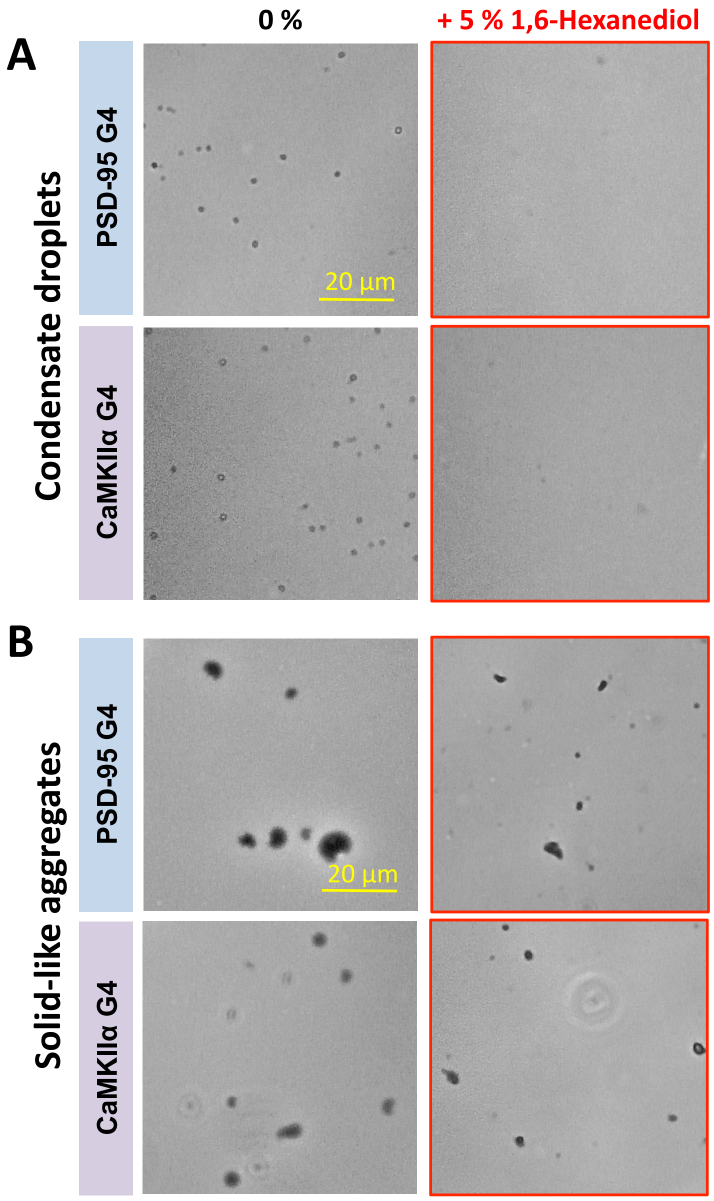

Figure. S9 1,6-Hexanediol sensitivity of condensate droplets and solid-like aggregates [Related to Figure 6].
Wild-type FUS (2 µM) was used to form G4-promoted condensate droplets and solid-like aggregates, respectively, and observed sensitivity to 5% 1,6-hexanediol by phase-contrast microscope. Although, most of condensate droplets formed by both PSD95-G4 and CaMKIIα-G4 disappeared in 15 minutes (*A*), solid-like aggregates were resistant to 1,6-Hexanediol (*B*).

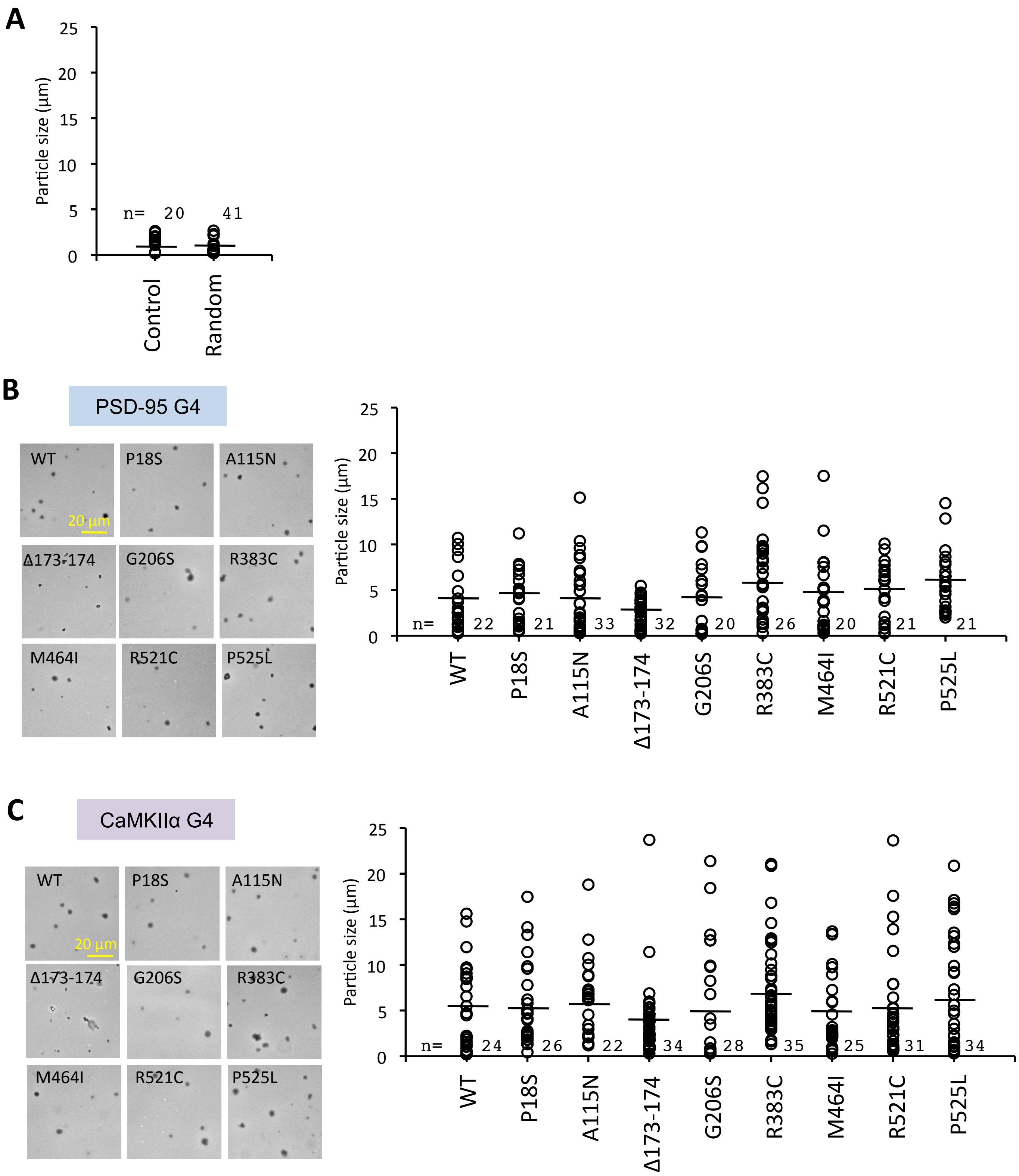

Figure S10. Determination of the size of mutant FUS compartments [Related to Figure 6].
Wild-type and eight mutant FUS proteins (2 µM each) were mixes with PSD-95 or CaMKIIα G4-RNA. The size of FUS condensate droplets was measured. *A*, Particle size of FUS condensates formed in the absence and presence of random RNA (2 µM). *B*, Particle size of FUS aggregates formed in the presence of 2 µM PSD-95 G4-RNA. *C*, Particle size of FUS aggregates formed in the presence of 2 µM CaMKIIα G4-RNA. The horizontal line shows the average value.

A

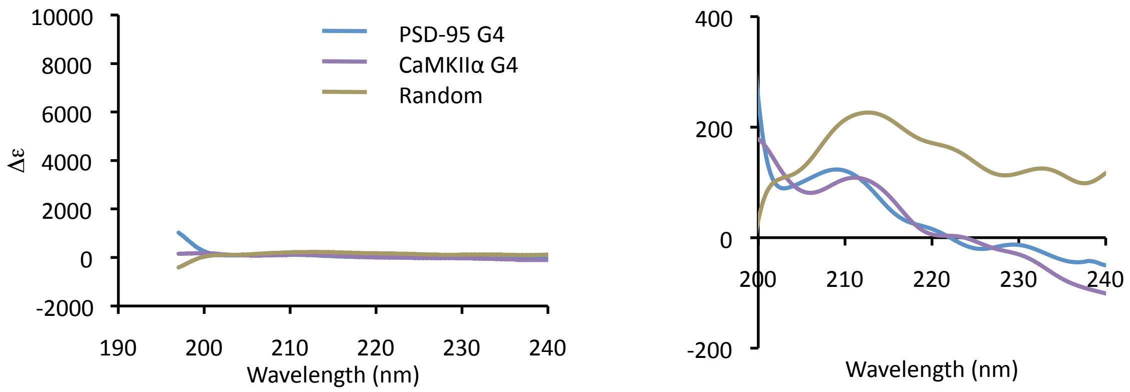

B

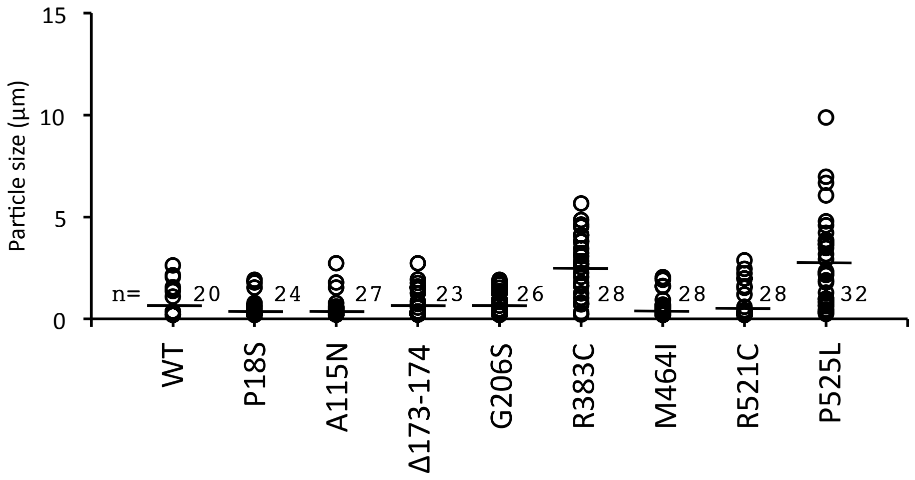

Figure S11. Structure-dependent changes of FUS mutant proteins [Related to Figure 7].
A, The CD spectra of RNAs in the absence of FUS protein. Right panel indicates higher magnification to show characteristic minimum around 240 nm. The characteristic spectral peaks of the protein do not overlap with RNA, suggesting that the error of concern is extremely small. In addition, spectral values were subtracted by each RNA spectrum to obtain an accurate protein spectrum. *B*, Particle size of wild-type and mutant FUS condensates (2 μM) formed in the absence of RNA.

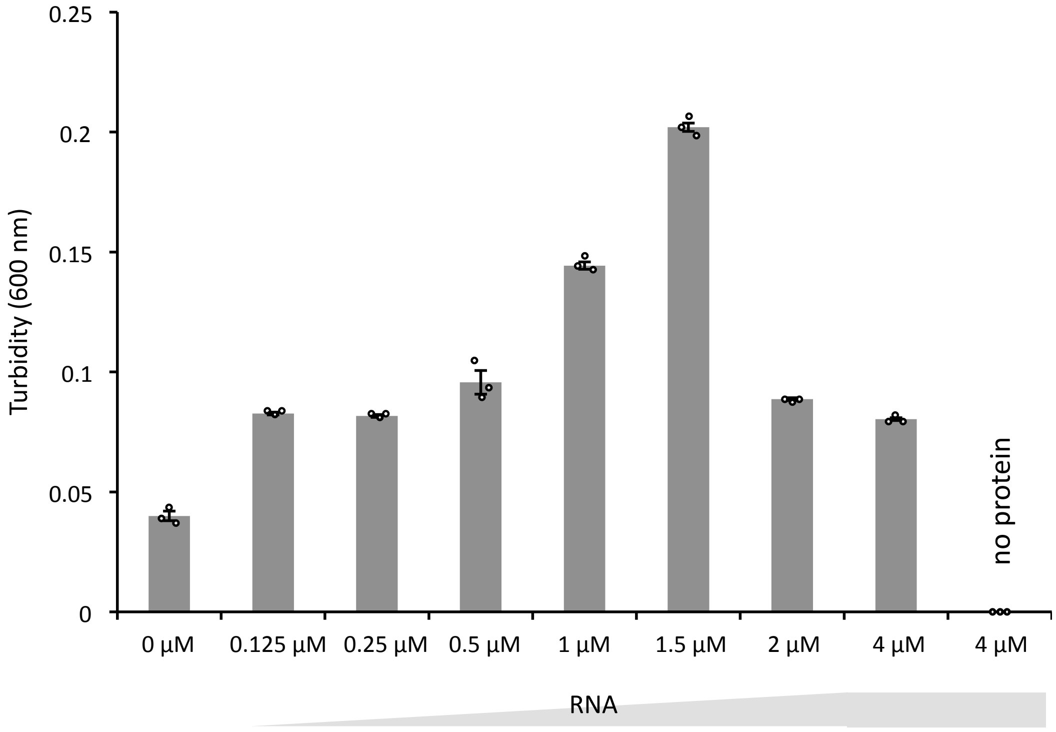

Figure S12. Phase separation assay with FUS and an increasing amount of the long promiscuous RNA. [Related to Figure 2C].

The 20 nt short randomized RNA did not inhibit LLPS, while the 68 nt long randomized RNA (8) did. This could be the dissolution of the condensate promoted by stoichiometry.

Table S1. Synthetic oligonucleotides used for this study.

Capital letters indicate deoxyribonucleotide and small letters indicate ribonucleotide.

Oligos	Nucleotide sequence (from 5' to 3')	
P18S-F	GGCCTACTCCACCCAGCCCGGGCAGG	
P18S-R	TGGGTGGAGTAGGCCCCATAGCTTTG	
A115N-F	TACGGTAACAGTTCTCAGAGCAGCAG	
A115N-R	AGAACTGTTACCGTAACTTCCCGAGG	
Δ173-174-F	AGGTGGAGGTAACTATGGCCAAGAT	
Δ173-174-R	AGTTACCTCCACCTCCACCTCCACC	
G206S-F	TGGCAGCAGTGGCTATGGACAGCAGGA	
G206S-R	TAGCCACTGCTGCCACCTCCACCACTC	
R383C-F	CAATGGTTGTGGAGGCCGAGGGCGAG	
R383C-R	CCTCCACAACCATTGCCACCACCCCG	
M464I-F	CTCACATCGGGGGTAACTACGGGGAT	
M464I-R	TACCCCCGATGTGAGAGCCACCTGGT	
R521C-F	ACAGGATTGCAGGGAGAGGCCGTATT	
R521C-R	TCCCTGCAATCCTGTCTGTGCTCACC	
P525L-F	GAGAGGCTGTATTAACTCGAGCACCA	
P525L-R	TTAATACAGCCTCTCCCTGCGATCCT	
PSD95 dA_{16}	ggggaaaagggagggauggg-dA_{16}	(Ishiguro et al., 2020)
CaMKIIα dA_{16}	ugggggggggcggguggga uggga-dA_{16}	(Ishiguro et al., 2020)
Telomere dA_{16}	GGGTTAGGGTTAGGGTTAGGGT-dA_{16}	(Ishiguro et al., 2021)
$(G4C2)_{4(R)}$ dA_{16}	ggggccggggccggggccggggcc-dA_{16}	(Ishiguro et al., 2021)
Random RNA (n20)	nnnnnnnnnnnnnnnnnnnn	
PSD-95	ggggaaaagggagggauggg	(Ishiguro et al., 2021)
Cy3-PSD-Ax_{647}	Cy3-ggggaaaagggagggauggg-Ax_{647}	(Ishiguro et al., 2021)
CaMKIIα	ugggggggggcgggugggauggga	(Ishiguro et al., 2021)
Cy3-CaMKII-Ax_{647}	Cy3-ugggggggggcggguggga-Ax_{647}	(Ishiguro et al., 2021)
