# Supplementary material for: ALS-linked FUS mutations dysregulate G-quadruplex-dependent liquid–liquid phase separation and liquid-to-solid transition
Source: J Biol Chem. 2021 Oct 6;297(5):101284. doi: 10.1016/j.jbc.2021.101284 (PMC8567205; doi:10.1016/j.jbc.2021.101284)
Supplement: Legend of Movie S1–S3 [file mmc5.docx]

**Legend of Supplemental movies**

**Movie S1. Real time image of LST through LLPS pathway [Related to Figure 6C].**

A mixture of wild-type FUS (2 μM) and PSD-95 G4-RNA (2 μM) was dropped onto the slide grass and the recording was started immediately at 25 °C.

**Movie S2. Real time image of LST through LLPS pathway [Related to Figure 6C].**

A mixture of wild-type FUS (2 μM) and PSD-95 G4-RNA (2 μM) was dropped onto the slide grass and the recording was started immediately at 25 °C. The condensate droplet collided with the FUS aggregates, leading to yield larger aggregate.

**Movie S3. Real time image of LST through LLPS pathway [Related to Figure 6C].**

A mixture of wild-type FUS (2 μM) and CaMKIIα G4-RNA (2 μM) was dropped onto the slide grass and the recording was started immediately at 25 °C. Adjacent condensate droplets collapsed in sequence, leading to yield larger aggregate.
